# Complex activity and short-term plasticity of human cerebral organoids reciprocally connected with axons

**DOI:** 10.1038/s41467-024-46787-7

**Published:** 2024-04-10

**Authors:** Tatsuya Osaki, Tomoya Duenki, Siu Yu A. Chow, Yasuhiro Ikegami, Romain Beaubois, Timothée Levi, Nao Nakagawa-Tamagawa, Yoji Hirano, Yoshiho Ikeuchi

**Affiliations:** 1 Institute of Industrial Science, The University of Tokyo, Meguro Tokyo, 153-8505 Japan; 2 Institute for AI and Beyond, The University of Tokyo, Bunkyo Tokyo, 113-8655 Japan; 3 Department of Chemistry and Biotechnology, The University of Tokyo, Bunkyo Tokyo, 113-8655 Japan; 4 LIMMS/CNRS, Institute of Industrial Science, The University of Tokyo, Tokyo, Japan; 5 IMS Laboratory, UMR5218, University of Bordeaux, Talence, France; 6 Department of Physiology, Graduate School of Medical and Dental Sciences, Kagoshima University, Kagoshima, Japan; 7 Department of Neuropsychiatry, Graduate School of Medical Sciences, Kyushu University, Fukuoka, Japan; 8 Department of Psychiatry, Division of Clinical Neuroscience, Faculty of Medicine, University of Miyazaki, Miyazaki, Japan

**Keywords:** Tissue engineering, Cellular neuroscience, Synaptic development, Short-term potentiation

## Abstract

An inter-regional cortical tract is one of the most fundamental architectural motifs that integrates neural circuits to orchestrate and generate complex functions of the human brain. To understand the mechanistic significance of inter-regional projections on development of neural circuits, we investigated an in vitro neural tissue model for inter-regional connections, in which two cerebral organoids are connected with a bundle of reciprocally extended axons. The connected organoids produced more complex and intense oscillatory activity than conventional or directly fused cerebral organoids, suggesting the inter-organoid axonal connections enhance and support the complex network activity. In addition, optogenetic stimulation of the inter-organoid axon bundles could entrain the activity of the organoids and induce robust short-term plasticity of the macroscopic circuit. These results demonstrated that the projection axons could serve as a structural hub that boosts functionality of the organoid-circuits. This model could contribute to further investigation on development and functions of macroscopic neuronal circuits in vitro.

## Abstract (web summary)

Connecting cerebral organoids with an axon bundle models inter-regional projections and enhances neural activity. Optogenetic stimulation induces short-term plasticity, offering insights into macroscopic circuit development and functionality.

## Introduction

A key structural feature that facilitates organized functional connectivity in the human brain is the macroscopic inter-regional connections that bridge physically separated and functionally distinct segments of the brain. Local neuronal circuits in distinct regions are inter-connected by axons extended from one region to another, which enables a whole brain to function coordinately and execute higher-order functions. With a delicate and dynamic balance, activity patterns of regional circuits interact with each other through the inter-regional connections, generating complex ensembles of oscillatory activity, and ultimately execute coordinated higher-order functions^1–3^. Malfunction and malformation of inter-regional connections contribute to various neuropsychiatric diseases including autism spectrum disorders, schizophrenia, and epilepsy^4–7^. Although it is evident that macroscopic inter-regional connections are essential for brain functions, the developmental and functional mechanisms of the connections are not yet fully defined due to the complexity of the circuits in the intact brain.

The macroscopic projections transmit action potentials and relay information from the sensory input to motor output, in which massively parallel signals are processed through many brain regions. In addition to the networks that convey information in a directional flow, loop structures can sustain and resonate information by “reentry” of neuronal activity and are considered to serve as fundamental mechanisms for temporal and sequential signal processing of the brain^8^. A loop circuit comprised of two cerebral regions can be established by bidirectional connections between two brain regions, which are observed and characterized as bidirectional axonal tracts in many parts of white matter in the brain. Despite their functional significance, development and functions of the reciprocal loop circuits have not been studied in detail in vitro due to lack of appropriate models.

Developmental and functional characteristics of neural networks can be investigated with great accessibility and manipulability in vitro. Recently, organoids have attracted attention due to their unprecedented potential for modeling human organs in vitro^9–11^. Neural organoids have been especially promoting human neuroscience research by providing a new path to overcome the difficulties of investigating human brains without damaging them. They mimic structural and cellular characteristics of human brain development, through in vivo-like differentiation and self-organization processes spontaneously executed by human stem cells’ intrinsic potential. Neural organoids have been successfully applied to model disorders including developmental defects, infection, psychiatric disorders, and neurodegenerative diseases^12–20^. Importantly, neural organoids exhibit spontaneous neuronal activity patterns resembling in vivo activity dynamics^21^. Cerebral organoids cultured for over six months showed oscillatory waves that resemble electroencephalography (EEG) activity patterns of the neonatal human brain^21^, which illustrated that neural organoids hold great potential to mimic human brain activities.

Most neural organoids model and recapitulate particular regions of the brain. Interactions between brain regions have been modeled by fusing two organoids mimicking distinct regions of the brain^22–25^. More complex circuits have been modeled by fusing multiple organoids^26^. The organoids successfully interact and mutually innervate; however, they lack white matter tracts between regions as observed in vivo. Due to its structural limitations, fusion of organoids does not allow axons to assemble bundles, a prominent feature underlying inter-regional functional connectivity in the human brain.

To understand how the inter-regional axonal tracts contribute to neuronal activity patterns, we analyzed neuronal activity of an organoid-based model system in which two cerebral organoids are bidirectionally connected by axons. This model exhibited intense and complex neuronal activity, demonstrating functional significance of the inter-organoid axonal connections. Optogenetic manipulations on the connecting axons demonstrated that oscillatory activity of the connected organoids could be entrained through synchronization with periodic stimulation. Induction of entrainment could be achieved with less stimulation after a repeated session, indicating that the first train of stimulations caused sustained changes on their later response to stimulations. These findings suggest that bidirectional axonal connections should be implemented in assembled models to enhance their functional resemblance to human brains.

## Results

### Neuronal activities of reciprocally connected cerebral organoids

**Fig. 1:**
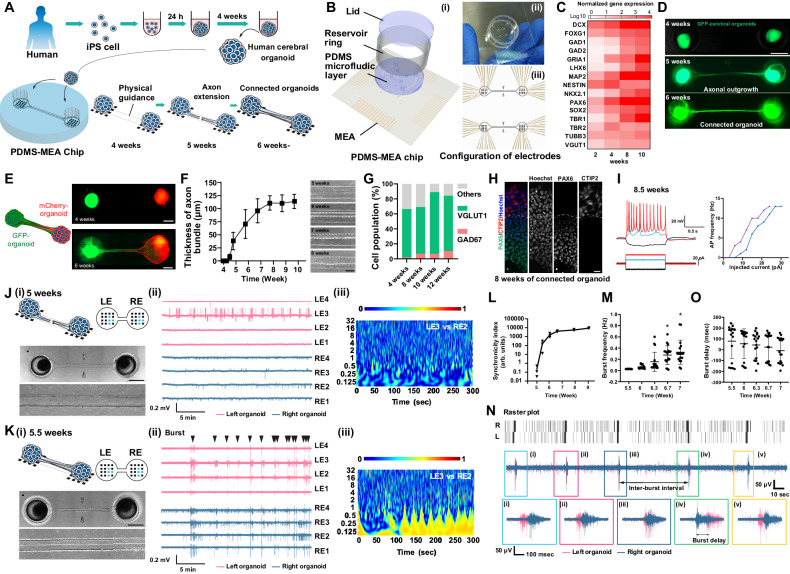
Formation and characterization of the connected organoids in a PDMS-MEA chip. **A** Preparation of the connected cerebral organoids. **B** PDMS-MEA chip schematic. **C** Gene expression profiles. Relative abundance of mRNAs was normalized to GAPDH. **D** Axons extended from one organoid to another organoid and formed axon bundles. Scale bar: 1 mm. **E** GFP-labeled and mCherry-labeled cerebral organoids were connected. Scale bar: 500 µm. **F** A time-course plot of axon bundle thickness. *n* = 7 (week 10) or 9 connected organoids (weeks 4–9). Scale bar: 100 µm. **G** The proportions of excitatory neurons, inhibitory neurons, and other neurons in the organoids. *n* = 3 organoids. **H** Immunohistochemical analyses revealed layers of different cell types within the connected organoids after 8 weeks of culture. Scale bar: 10 µm. **I** Whole-cell patch-clamp recording in neurons in connected organoids after 8.5 weeks from differentiation when hyperpolarizing and depolarizing square wave current pulses were injected. Action potential frequency responded with the different injected current (0 ± 30 pA, 1 s). **J** (i) Representative images of connected organoids after 5 weeks of culture. Scale bar: 1 mm. (ii) Filtered signals from four representative electrodes under each organoid. (iii) Wavelet coherence between signals from electrodes of each organoid in the connected organoid. **K** Representative images, filtered signals, and wavelet coherence of the connected organoids after 5.5 weeks of culture. (ii) Black arrowheads represent synchronized burst activities associated with dense spikes. (iii) The wavelet coherence indicated a strong correlation between the two connected organoids. **L** Synchronicity of activity in the two connected organoids increased during the culture period. *n* = 4 organoids. **M** Burst frequency increased significantly with culture time. *n* = 12 organoids (week 5.5) or 18 organoids (week 6, 6.3, 6.7 and 7). *P* = 2.4e−4; 7.7e−6 (6.7 weeks and 7 weeks relative to 5.5 weeks). **N** Magnified view of the plot of neuronal activity of the two connected organoids. Synchronized burst activity was observed with a delay. **O** Burst delay after different culture periods. *n* = 20 bursts. *p < 0.05; one-way ANOVA with Tukey’s multiple comparison. LE left electrode, RE right electrode. Data are presented as mean values ± SD.

Cerebral regions reciprocally connect with each other through axonal tracts to form a “reentry” circuit which is one of the simplest, yet functionally important macroscopic loop architecture in the brain^8^. The reciprocal extensions of axons interact in a “hand-shaking” manner that could guide the axons mutually to the targets^27^. We have previously reported a culture model system to structurally mimic the inter-regional cortical connections by culturing a pair of human induced pluripotent stem (iPS) cell-derived cerebral organoids^28^, however their functionality had not been assessed. To analyze their neuronal activity, we have developed a culture chip equipped with electrodes (Fig. 1A, B). The chip (PDMS-MEA chip) consisted of a multielectrode array (MEA) layer and polydimethylsiloxane (PDMS) microfluidic layer. The PDMS layer possessed two holes, each of which received a cerebral organoid. The two holes were connected by a channel providing spatial guidance for axons to reciprocally target the organoid at the opposite end of the chip. We observed an increase in the expression of neural stem cell and neuronal markers (e.g., PAX6, SOX2, DCX) and cortical layer-associated genes (e.g., TBR1) over 2–10 weeks of culture of cerebral organoids generated from iPS cells (409B2), confirming the differentiation to cortical neurons (Fig. 1C). The organoids were placed into the PDMS-MEA chip at 4 weeks. At 6 weeks (2 weeks in the chip), the two cerebral organoids formed an axon bundle which connected the organoids in the chip (Fig. 1D). GFP-expressing and mCherry-expressing cerebral organoid demonstrated the hand-shake-like connectivity in the chip (Fig. 1E, Supplementary Fig. S1A–H). The thickness of the axon bundle gradually increased up to 120 µm until 8 weeks of differentiation (Fig. 1F) and did not contain any cell bodies (Supplementary Fig. S1I, J). VGLUT1-positive excitatory neurons and GAD67-positive inhibitory neurons comprised approximately 60–80% and 5–10% of cells in the organoids, respectively (Fig. 1G). Similar developments in axon bundle formation and cell type distribution could be observed when generating connected organoids with a different cell line (Supplementary Fig. S1K–N). Subcortical layer-like structures were preserved in the connected organoids (Fig. 1H). Electrophysiological properties were analyzed from the individual neurons in connected organoid after 8.5 weeks from differentiation by whole-cell path-clamp recording (Fig.1I and Supplementary Fig. S2A). Mature neurons which exhibited sustained and sharp peaks of action potentials were found, while immature neurons which showed weak responses were also observed (Supplementary Fig. S2B–D). Mature neurons in the connected organoids generated more action potentials as injected current was increased (Fig. 1I and Supplementary Fig. S2D), and also exhibited spontaneous firings (Supplementary Fig. S2E).

We then recorded neuronal activities of the connected organoids with MEA (Supplementary Fig. S3A). MEA positioned underneath the two organoids allows us to capture neuronal activity in the PDMS device (Fig. 1B). In addition to action potential spikes of individual neurons, the electrodes can detect so called “local field potentials (LFPs)” which are generated from ensemble of clustered activities of neurons nearby the electrode^29^. The action potential spikes and LFPs were extracted by a high-frequency filter (300–3000 Hz) and a low-frequency filter (>1000 Hz), respectively. First, we assessed the time-course development of the electrical activity of the organoids in the chip. After 4.5–5 weeks (0.5–1 week of culture in the chip), electrical activity was detected from the organoids (Fig. 1J). At this stage, sparse action potential spikes were detected from the organoids, but the neuronal activity of the two cerebral organoids was not correlated. The lack of correlation between the organoids was consistent with the lack of axonal connections between the organoids. At 5.5–6 weeks (1.5–2 weeks of culture in the chip), we observed synchronized burst activity which lasts for more than 100 msec and the ensemble signal was observed in low frequency (<1 Hz) range (Fig. 1K, black arrow). The escalation and synchronization of the activity coincided with the physical connectivity of the organoids via an axon bundle. From 5 to 7 weeks of culture, neural activity became more synchronized, and the burst activity became more frequent (Fig. 1L, M). The thickness of axon bundles exhibited positive correlation with frequent neuronal activity (Supplementary Fig. S3B). Meanwhile, we did not observe a change in spike and burst frequency upon variation of axon bundle length (Supplementary Fig. S3C). Notably, a small temporal shift (around or less than 100 msec) between a burst signal from an organoid and that from another organoid was observed (Fig. 1N, O). The two organoids frequently alternated to initiate signal propagation, indicating that the axonal macroscopic connections were functionally bidirectional. Though bursts of one organoid often proceeds those of another, the average delay of left-proceeding bursts and right-proceeding bursts were comparable (Fig. 1O, 100–200 msec), suggesting that propagation velocity is similar between both directions. The directionality of the burst propagation may represent a slight temporal shift (e.g. intrinsic variability) of the two connected organoids. Interestingly, the bursts could also occur almost at the same time in the two organoids without or with a little delay (<50 msec). This suggests that the activity of the two organoids sometimes could be in “harmonic” synchronization. These suggest that the connected organoids might be generating complex activity patterns through both mutual propagation and synchronization of burst activity.

### Inter-organoid axon bundles induced neuronal activity

**Fig. 2:**
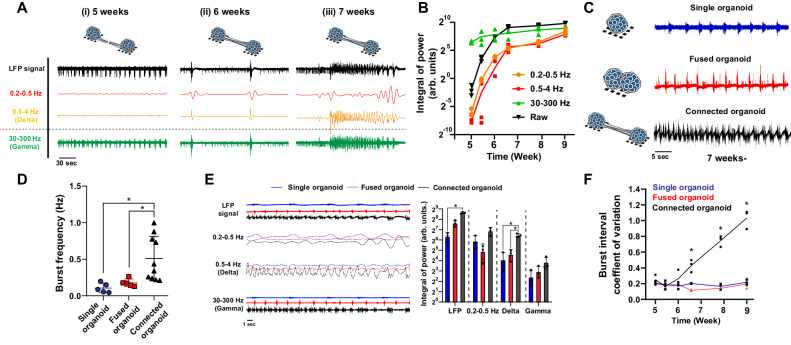
Reciprocal connections through bundled axons generate complex neuronal activity. **A** LFP signals were extracted from the 0.2–0.5 Hz, 0.5–4 Hz (delta), and 30–300 Hz (gamma) bands by inverse continuous wavelet transformation. At 8 weeks, the connected organoids generated slow-wave oscillations in the 0.5–4 Hz (delta) band. **B** Integral of power in frequency bands. *n* = 3 organoids. **C** Representative neuronal activity of the single, fused and connected organoids. **D** Burst frequency of the single, fused and connected organoids. *n* = 5 organoids (single and fused), 10 organoids (connected). *P* = 0.0123 (single/connected); 0.0171 (fused/connected). **E** Inverse continuous wavelet transformation in the 0.2–0.5 Hz, 0.5–4 Hz (delta), and 30–300 Hz (gamma) bands. Delta band oscillations were observed in the connected organoids but not in the single or fused organoids. *n* = 3 organoids. *P* = 0.0242 (LFP, single/connected); 0.0054 (delta, single/connected); 0.0208 (fused/connected). **F** Inter-event interval coefficient of variation among the three types of organoids. *n* = 3 organoids. *P* = 0.0019 (6.5 weeks, single/connected); 0.0003 (6.5 weeks, fused/connected); 1.2e−5 (7.8 week, single/connected); 9.1e−6 (7.8 week, fused/connected); 4.4e−5 (9 week single/connected); 3.8e−5 (9 week, fused/connected). *p < 0.05; One-way ANOVA with Tukey’s multiple comparison test. Data are presented as mean values ± SD.

Next, we characterized developmental time-course of LFP signals of the connected organoids. At 6 weeks (2 weeks of culture in the chip), slow LFP signals (0.2–0.5 Hz) were observed in the connected organoids (Fig. 2A), which were absent prior to the establishment of axonal connections at 5 weeks. After another week in the chip (7 weeks of culture), the LFP became more complex, and intense activity in the ‘delta’ frequency band (0.5–4 Hz) emerged (Fig. 2A, B), which indicates that coordinated ensemble of neuronal activity was developed. Magnitude of LFP increased over time in multiple frequency bands (Fig. 2B). The intense and complex activity of the connected organoids at this relatively early stage of culture was unexpected considering a previous report in which cerebral organoids exhibited LFP in the delta band after culture for a few months^21^.

To assess the functional significance of the inter-organoid connection through an axon bundle, we compared the neuronal activity of the ‘connected’ organoids with that of conventional cerebral organoids (‘single’ organoids). The connected organoids contained approximately twice the number of cells of a single organoid, which could affect neuronal activity. We thus also assessed the activity of tissues generated by directly fusing two cerebral organoids (‘fused’ organoids) as an additional control (Fig. 2C and Supplementary Fig. S3E–G). After 6 weeks of culture, periodic burst activity was detected in all of them. Notably, the connected organoids exhibited significantly more frequent burst activity than the single or fused organoids (Fig. 2D), although the numbers of neurons in the fused and connected organoids were comparable (Supplementary Fig. S3H). These findings suggest that the inter-organoid axonal connections enhanced neural activity. To understand the network connectivity of the organoids, we analyzed signal propagation velocity (Supplementary Fig. S3I–K). The signal propagation speed between the connected organoids was faster than that within an organoid. In contrast, the inter-organoid signal propagation velocity in the fused organoids was significantly less than that within a single organoid. In fact, inter-organoid signal propagation lag was significantly bigger in the fused organoids than in the connected organoids, suggesting that the axon bundle provides faster and stronger communication between organoids than the interface of the directly fused organoids.

We then compared the neuronal activity of the single, fused, and connected organoids in more detail. The connected organoids exhibited significantly richer activity in the delta frequency band than the single or fused organoids (Fig. 2E). The coefficient of variation (CV) of burst interval was significantly higher in the connected organoids than in the single or fused organoids (Fig. 2F), indicating that bursts were significantly less periodic and more intricate in the connected organoids than in the single or fused organoids. The CV increased consistently over 9 weeks of culture in the connected organoids but did not change significantly in the single organoids or the fused organoids during the observed period. Similar activities were seen when recording from connected organoids generated from another iPS cell line (30HU-002, Supplementary Fig. S4A–I). These results suggest that the reciprocal connections induced complex activity in the connected cerebral organoids.

### Axon bundle-associated neurons exhibit expression signature of activation and maturation

To understand the structure and morphology of axons extended into the bundle, we performed labeling experiments. We employed “Brainbow” method to visualize neurons and their axons within the connected organoids (Supplementary Fig. S5). Although the labeling was successful, the density of neurons and axons in the tissues was too high to trace axons to precisely locate their cell bodies. We also performed single cell (sc) RNA-seq on the single, fused, and connected organoids at 7 weeks of culture (Supplementary Fig. S6A, B), which revealed overall similarity in gene expression profiles of the organoids with small differences (Supplementary Fig. S6C, D). Pseudo-time analysis revealed that cluster 8 contains most mature cells within the cerebral organoids (Supplementary Fig. S6E). The cells in the cluster 8 abundantly express a set of genes that are important for neuronal development (Supplementary Fig. S6F, G), suggesting that the connected organoids might possess neurons that are more developed than single or fused organoids.

**Fig. 3:**
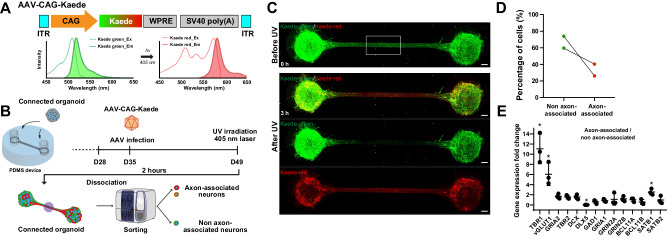
Photo-convertible fluorescent revealed the important role of axon bundle and their specific population. **A** Plasmid construct expressing photo-convertible fluorescent protein Kaede, under CAG promotor in pAAV backbone plasmid. Kaede green fluorescent protein can be converted to Kaede red fluorescent protein by UV exposure. **B** AAV-CAG-Kaede was infected one week after introducing cerebral organoid into microfluidic device. At day 49 (7 weeks of culture), UV light (405 nm laser equipped with a confocal microscope) was irradiated to the axon bundle. Then, the cells were sorted by cell sorter to identify axon bundle-associated neurons (Kaede-red positive) and non-associated neurons (Kaede-red negative). **C** Kaede photo-conversion in connected organoids before and after UV light-irradiation. UV exposure rapidly converted Kaede-green to Kaede-red, then, Kaede-red was quickly diffused in the axon bundle to both anterograde and retrograde direction, resulting in the gradation of Kaede-green and Kaede-red in axon bundle. Scale bar: 150 µm. **D** The ratio of axon bundle-associated neurons and non-associated neurons from two independent samples. The average percentage of axon bundle-associated neurons was 32%, whereas that of non-associated neurons was 68%. **E** Relative fold change of gene expressions in axon bundle-associated neurons to non-associated neurons. TBR1 and VGLUT1 were highly expressed in axon bundle-associated neurons. *n* = 3 organoids. *P* = 0.0004 (TBR1 relative to GAPDH); 0.0168 (VGLUT1); 8.5 e–10 (DLX5); 0.0117 (SATB1). *p < 0.05, Student’s *t* test (two-sided). Data are presented as mean values ± SD.

A green-to-red photo-convertible fluorescent protein Kaede^30^ was employed to identify neurons extending their axons into the bundle (Fig. 3A, B). Before UV exposure, Kaede-green was distributed in the entire connected organoids, but little Kaede-red was present (Fig. 3C). After UV light-irradiation to the center of axon bundle, we let the converted Kaede-red proteins migrate to the cells for additional 2 h, which resulted in successful visualization of axon bundle-associated neurons in the connected organoids (Fig. 3C). Flow cytometry analysis revealed that approximately 30% of neurons extended their axons into the bundle between the organoids (Fig. 3D). The expression levels of TBR1, and VGLUT1 were significantly higher in axon bundle-associated neurons than non-associated neurons (Fig. 3E). These data suggest that the inter-organoid axons in the bundles are associated with relatively mature neurons, and the synaptic connections could promote their maturation, which in turn could contribute to the advanced neuronal activity of connected organoids.

### In silico modeling of the connected organoids

To analyze if structure of organoids can alter their activity patterns, we generated an in silico model using a Hodgkin-Huxley formalism (Supplementary Fig. S7). This in silico model of organoids consists of regular spiking neurons (RS) as excitatory neurons and fast spiking neurons (FS) as inhibitory neurons (Supplementary Fig. S7A, B). The network structures of the single, fused, and connected organoids were simplified and modeled by the in silico neuron models (Supplementary Fig. S7C–E). To model the inter-organoid connections, we optimized the synaptic weights of the inter-organoid connections. We found that we could mimic the electrical activity of the connected organoids by setting higher synaptic weight for the inter- than the intra-organoid connections, with which the connected organoids exhibited increased burst frequency than single and fused organoids, consistent with the actual in vitro recordings (Supplementary Fig. S7F, G). This indicates that the intense activity of the connected organoids could be generated through robust synaptic connections between the organoids.

### Optogenetic inhibition of the inter-organoid axons

**Fig. 4:**
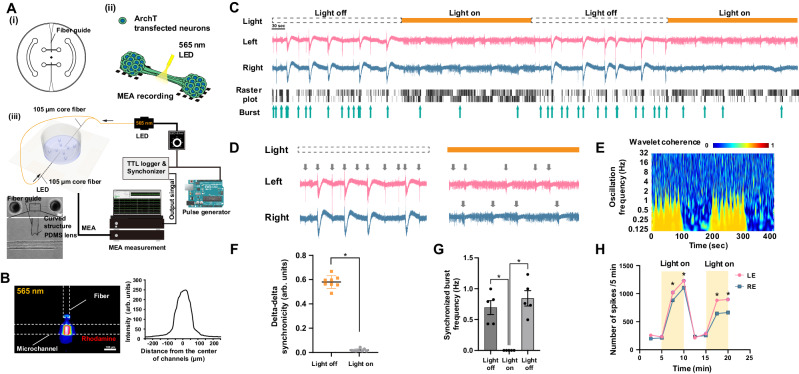
Optogenetics inhibition and synchronicity of burst activity between organoids. **A** Optogenetic setup for inhibiting the synaptic interaction between left and right organoids through axon bundle. (i) A microfluidic device for optogenetic control consisting of fiber guides (thin channels) for optical fiber insertion. (ii) ArchT was expressed in the connected organoid by AAV. The light at 565 nm waveband illuminated the axon bundle in microfluidic device. (iii) An optical fiber was connected to 565 nm LED and a pulse generator (Arduino). An optical fiber was positioned perpendicular to the axon bundle with a 100 μm gap. Light exposure timing and a representative channel from MEA amplifier were recorded in TTL logger to synchronize TTL signal and recording. **B** An axon bundle and an optical fiber. Curved structure serves as a PDMS lens which helps the light to be focused on the axon bundle. **C** LFP and raster plot from left (pink) and right (blue) organoids of a connected organoid with or without light illumination (orange bar: TTL). **D** Synchronized burst frequency was around 0.65 Hz in the absence of light illumination and decreased to zero during light on. Then, synchronized burst frequency immediately increased and recovered after stopping the light. **E** Wavelet coherence by wavelet transformation revealed that slow wave oscillation disappeared during light illumination, indicating the vanishment of any correlative activity during light exposure. **F** Light illumination completely suppressed these synchronized burst activities. *n* = 8 from 3 organoids. *P* = 1.5e−8. **G** Inter-regional synchronicity measured by delta phase-delta phase coupling showed significant decrease during light illumination. *n* = 5 from 3 organoids. *P* = 0.0008 (first light off/light on); 0.0001 (second light off/light on). **H** Total numbers of single spikes were calculated in a 5 min time slot. Light-on significantly induced the increase in number of spikes. *p < 0.05; Paired-test (two-sided) for **F** and one-way ANOVA with Tukey’s multiple comparison for **G**. Data are presented as mean values ± SD.

To interrogate whether axonal connections facilitate the complex activity of the connected organoids, we performed loss-of-function experiments by inhibiting the axonal transmission between the organoids. Physical severance of the axon bundle between organoids disrupted the bursts and LFP patterns in the delta band (Supplementary Fig. S8). To further assess the role of the axonal connections between the organoids, we optogenetically inhibited the axon bundle (Fig. 4A). The axon bundle between the organoids provides excellent accessibility for light exposure in optogenetic neural manipulations. To achieve light-dependent inhibition of the neuronal activity of the organoids, ArchT, an orange light-dependent outward proton pump^31^, was expressed in organoids. With an optic fiber and a PDMS lens, the light was irradiated only on the axon bundle in the microchannel (Fig. 4B). Illumination of the axon bundle with orange light (565 nm, 20 msec, 20 Hz for 5 min) resulted in loss of high-amplitude bursts and low-frequency delta-LFP patterns (Fig. 4C, D), suggesting that action potentials traveling through the axon bundle induce the burst activity in the connected organoids. Upon cessation of illumination, the burst activity and delta-LFP patterns were immediately restored. These light-induced responses were repeatedly observed after light exposure. The frequency of spontaneous bursts did not change after stopping the light exposure, suggesting that intrinsic internal circuit properties determined the frequency of the burst activity. Notably, the coherence and synchronicity of the signals from the two connected organoids were significantly decreased by the light exposure (Fig. 4E, F). While bursts disappeared upon optogenetic inhibition of inter-organoid axons, the number of observed action potentials increased (Fig. 4G, H). This observation is consistent with the observation that the axon bundle-associated neurons were mostly excitatory neurons (Fig. 3E). These results indicate that inter-organoid axons contributed to generating bursts by temporally orchestrating and aggregating the activity of individual neurons. These results indicated that activity transmitted via the inter-organoid axon bundle underpinned the complex neuronal activity in the connected organoids, modeling the development and functionality of macroscopic connections in the brain.

### Network activity characteristics of the connected organoids

**Fig. 5:**
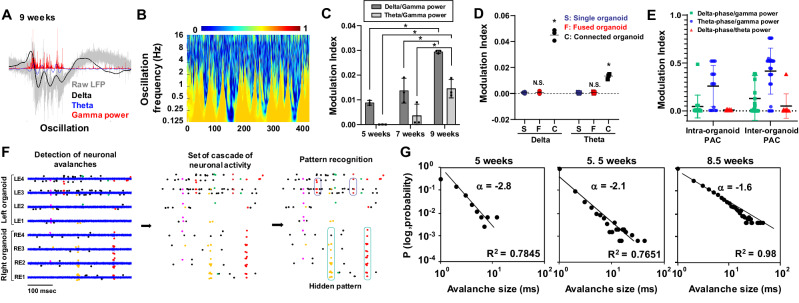
Theta-band oscillation, phase-amplitude coupling, and neuronal avalanches indicate complex activity in the connected organoids. **A** Raw LFP plot from four electrodes of each of the connected organoids at 9 weeks. **B** Wavelet coherence between the two organoids exhibited synchronous activity in the theta band frequency. **C** Modulation index of PAC in delta-phase/gamma-power and theta-phase/gamma-power of the connected organoids cultured for 5, 7, and 9 weeks. *n* = 3 organoids. *P* = 0.0004 (delta, 5–9 weeks); 0.0016 (delta, 7–9 weeks); 0.0052 (theta, 5–9 weeks). **D** Delta-phase/gamma-power and theta-phase/gamma-power PAC modulation index of single, fused, and connected organoids. *n* = 3 organoids. *P* = 8e−7 (delta, single/connected); 9e−7 (fused/connected); 7.35e−5 (theta, single/connected); 0.0002 (fused/connected). **E** Intra- or inter-organoid PAC modulation index in the connected organoids. *n* = 16 from 4 organoids. **F** Schematic illustration of analyses of neuronal avalanches including the extraction of neuronal avalanche cascades. Neuronal avalanches were calculated from 8 electrodes. The cascade of single spikes was characterized at 3 msec scale size. **G** The log plot of neuronal avalanche size and probability at 5, 5.5, and 8.5 weeks of culture. *p < 0.05; One-way ANOVA with Tukey’s multiple comparison test. Data are presented as mean values ± SD.

Extension of the culture period to 8–9 weeks resulted in a further increase in LFP frequency and action potential spikes of the connected organoids (Fig. 5A, Supplementary Fig. S9A). The complexity of the signals also increased, and rich ‘theta’ band (4–8 Hz) activity was observed. The representative plot depicts association of gamma activity with delta and theta activity (Fig. 5A, B, Supplementary Fig. S9A, B). To examine the relationship between low-frequency activity and the amplitude of high-frequency spikes, we evaluated phase-amplitude coupling (PAC)^32,33^ of the connected organoids. Delta-gamma PAC intensified with culture time, followed by emergence of theta-gamma PAC (Fig. 5C, Supplementary Fig. S9C). Modulation index of both delta-gamma and theta-gamma PAC in the connected organoids was significantly higher than those of the single or fused organoids (Fig. 5D, Supplementary Fig. S9D). PAC is believed to be a fundamental mechanism for functional interaction between brain regions. To understand the network activity characteristics of the two connected organoids, we examined inter-organoid PACs. Delta-gamma and theta-gamma inter-organoid PAC was stronger than that in intra-organoid PAC (Fig. 5E), indicating robust communication between the two organoids contributes to their orchestrated delta and theta band activity.

We performed Ca^2+^ imaging on the connected organoids in combination with MEA recordings (Supplementary Fig. S10A, B), which demonstrated consistency between MEA signals and Ca^2+^ surge in the organoids (Supplementary Fig. S10B, C). During a burst activity, neurons within the same organoid exhibited strong temporal correlation, but inter-organoid correlation was weak (Supplementary Fig. S10D, E). When activity of neurons in one organoid was shifted by 50 msec, we could observe strong inter-organoid correlation, consistent with the delay observed by MEA (Fig. 1N). The correlation varied between neurons, suggesting that evaluating activity dynamics of individual neurons could provide more information of the network in future studies.

To understand network activity patterns of connected organoids, we examined ‘neuronal avalanches’, which are trains of temporally proximal signals observed within 3 msec apart from each other across the electrodes (Fig. 5F). The neuronal avalanches are “critical phenomena” of chaotic neuronal network activity, and their size exhibits power law distributions if the network is adequately assembled and matured^34–36^. The power law exponent of neuronal avalanches represents the critical dynamics and synaptic connectivity of neuronal networks. Consistently, avalanche size distributions changed as the connected organoids were cultured. At 5 weeks, the connected organoids exhibited an exponent of α = −2.8 (Fig. 5G). As the organoids grew axons into the channel and formed connections at 5.5 weeks, the exponent increased to –2.1. At 8.5 weeks, the exponent became α = –1.6. The constant increase of the neuronal avalanche power exponent indicated that the neuronal networks in the connected organoids were gradually assembled and matured during the culture period. The value at 8.5 weeks (−1.6) was close to the theoretical exponent of –1.5 in the critical branching process^36^, suggesting that the neural circuits in the connected organoids are well assembled. This increasing trend was consistent in another line (30HU-002)-derived connected organoids (Supplementary Fig. S9E).

Collectively, the connected organoids exhibited patterns of complex activity which suggest the enriched network structure and function in the tissue.

### Optogenetic entrainment of the connected organoids

**Fig. 6:**
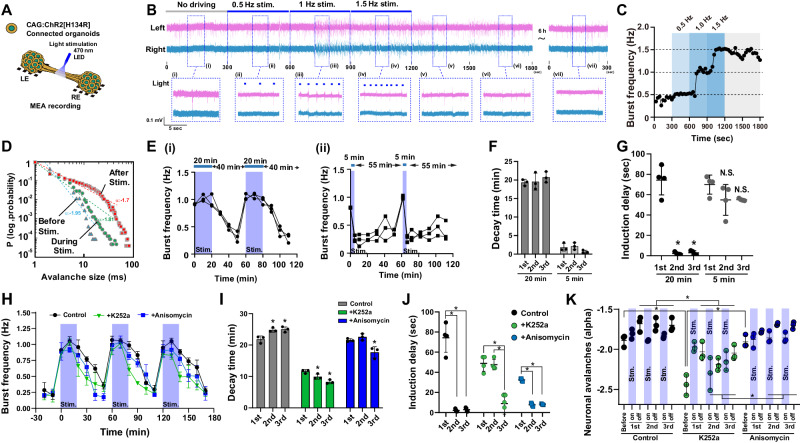
Potentiation of the connected organoids by optogenetic stimulation. **A** Optogenetic setup for stimulation of the axon bundle. **B** Optogenetic stimulation drove synchronized burst activity. **C** Burst frequency was modulated by optical stimulation. The burst frequency followed the stimulation frequency after a significant delay. **D** Log plot of neuronal avalanche size and probability before, during, and after stimulation. **E** Time course of burst frequency with 1 Hz stimulation for (i) 20 min or (ii) 5 min every hour. *n* = 3 organoids. **F** Duration until the burst frequency decreased to 75% of the maximum burst frequency after cessation of light stimulation. *n* = 3 organoids**. G** The delay from the start of light stimulation to the induction of burst frequency was significantly reduced during the second and third attempts when the connected organoids were stimulated for 20 min. *n* = 4 organoids. *P* = 2.1e−6, 2.3e−6 (2nd and 3rd relative to 1st, within 20 min stimulation). **H** Time series of burst frequency in the presence of K252a or anisomycin. *n* = 3 organoids. **I**, **J** Duration of sustained activity (**I**) (*n* = 3 organoids. *P* = 0.0123; 0.0096 (2nd and 3rd respectively, in comparison with 1st within control); 0.0435; 0.0019 (2nd and 3rd respectively, in comparison with 1st within K252a); 0.0153 (3rd, in comparison with 1st within anisomycin)) and the delay of burst frequency induction (**J**) of the connected organoids in the presence of K252a or anisomycin, as shown in **F** and **G**. *n* = 4 organoids. *P* = 2.1e−6, 2.3e−6 (control); 3.3e−5, 4.5e−5 (K252a); 2.4e−9, 2.7e−9 (anisomycin). **K** Probability slope of neuronal avalanches. K252a treatment, but not anisomycin treatment, led to a decreased probability of neuronal avalanches. *n* = 3 organoids. *P* = 2.8e−17 (control/K252a before); 3.9e−6 (control/K252a 2nd off); 6.2e−7 (K252a/anisomycin before); 3.7e−5 (K252a/anisomycin 2nd on); 2.1e−8 (K252a/anisomycin 2nd off); *P* = 0.0025 (control/K252a 1st off); 0.002 (control/K252a 2nd on); 0.0144 (control/K252a 3rd on); 0.0031 (control/K252a 3rd off); 0.0315 (K252a/anisomycin 3rd on); 0.0025 (K252a/anisomycin 3rd off). *p < 0.05; one-way ANOVA with Tukey’s multiple comparison test. Data are presented as mean values ± SD.

To characterize evoked responses of the connected organoids to external stimulations, we again employed an optogenetic approach using channelrhodopsin (ChR2[H134R]) (Fig. 6A). We stimulated ChR2-expressing connected organoids with pacing illumination (470 nm, 200 msec) at the axon bundles at the same rate of the spontaneous bursts (0.5 Hz), which did not alter the burst frequency. Then, they were stimulated with higher frequency at 1.0 Hz for 5 min, followed by 1.5 Hz for 5 min (Fig. 6B). Bursts were increased in accordance with the stimulation frequency. After cessation of stimulation, the frequency of the bursts was elevated for a while but eventually returned to the pre-stimulation frequency (Fig. 6B, C). The sustained elevated frequency of burst activity indicated that the temporal activity patterns of the connected organoids could be modulated by external stimulation and maintained for a certain period, demonstrating that the connected organoids exhibit short-term plasticity as a tissue of neuronal network.

Notably, the light stimulation did not induce bursts instantly. Instead, it induced bursts with a delay (“induction delay”, about 80 s) after the stimulation started (Fig. 6C), suggesting that repeated stimulations were necessary to modulate the activity of the connected organoids. During this delay, the neuronal avalanches were extended (Fig. 6D), suggesting that the neurons in the connected organoids slowly adapted to the stimuli and are gradually recruited to the potentiated and readily excitable subcircuits before the connected organoids fully followed the temporal patterns of external stimulation.

Stimulation of connected organoids at 1 Hz for 20 min induced burst activity (Fig. 6E, F). The burst frequency was higher than the baseline for about 20 min after the stimulations (“decay time”). In contrast, shorter stimulation (5 min) did not induce sustained burst activity, although burst frequency was elevated during the stimulation. This suggests that the circuit-level potentiation depends on the stimulation dosage.

After the burst frequency was returned to normal from the stimulation-induced elevated levels, we stimulated the connected organoids again. Interestingly, the induction delay for adaptation to the stimulus was significantly shorter on the second and third stimulation sessions than on the first stimulation for 20 min (Fig. 6G). The induction delay did not alter when the connected organoids were repeatedly stimulated for 5 min. These indicated that the connected organoids exhibit network-level potentiation upon stimulation, which can be maintained internally even after their activity apparently returned to the baseline.

We performed the entrainment experiment with connected organoids generated from another iPS cell line (30HU-002, Supplementary Fig. S11A). We observed the induction delay for the first train of stimulation until the activity followed the stimulation pattern. The induction delay was significantly reduced in the second and third stimulation session, consistent with what we previously described (Supplementary Fig. S11B). Meanwhile, we did not observe the sustained activation after stopping the stimulations, indicating that the cell line-to-cell line variability has profound effect on the post-stimulation behavior of neurons in the organoids.

Neuronal plasticity is regulated through diverse molecular programs, including calcium-dependent signaling pathways and local protein synthesis, essential for the early and late phase of the response, respectively^37–39^. To probe the mechanisms underlying potentiation in the connected organoids, we treated the organoids with K252a, an inhibitor of the key calcium signaling proteins CaM kinases, and anisomycin, an inhibitor of protein synthesis, during the optogenetic stimulation experiment (Fig. 6H, Supplementary Fig. S11C). The connected organoids responded to optical stimulation, and the sustained activity after stimulation was observed upon treatment with K252a or anisomycin. The decay time of the post-stimulation sustained activity significantly decreased in the presence of K252a after the second and third stimulations (Fig. 6I). In contrast, anisomycin treatment resulted in a slight decrease in the sustained activity after only the third stimulation. After the second stimulation, K252a treatment inhibited the shortening of the induction delay between the start of stimulation and the response of the connected organoids compared to that of the control (Fig. 6J and Supplementary Fig. S11D). Furthermore, the probability of neuronal avalanches decreased with K252a treatment, but was unchanged upon anisomycin treatment (Fig. 6K and Supplementary Fig. S11E). These results indicated that calcium-dependent signaling pathways underpin the short-term potentiation observed in our experiments.

We also stimulated one of the two connected organoids instead of the axons connecting them (Supplementary Fig. S12A). The stimulation successfully induced entrainment of the activity of the connected organoids, but we did not observe induction delay (Supplementary Fig. S12B, C). This suggests that the stimulation on an organoid could activate a larger population of neurons than the stimulation on axons, and the stimulation was too strong to appreciate the increase of induced activity. Similarly, no induction delay was observed when single organoids were stimulated (Supplementary Fig. S12D–G) for the same reason. To examine if a burst magnitude could impact the propagation delay of the burst to the connected organoid, we stimulated one of the two connected organoids with different stimulation strengths (Supplementary Fig. S13). The strength of stimulation did not alter the burst propagation delay, although the initial induction of a burst in the stimulated organoid was faster with stronger stimulation.

**Fig. 7:**
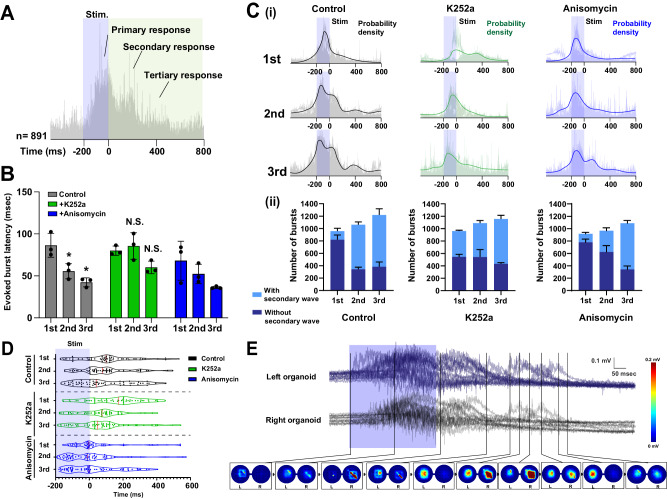
Evoked bursts self-reinforce the diversity of burst activity in a CaM kinase-dependent manner. **A** Representative image of sorted bursts evoked by optogenetic stimulation. A total of 891 burst traces are presented. Light stimulation induced evoked spikes, and the burst responses persisted after light stimulation. Secondary and tertiary responses were also observed. **B** Latency of the evoked bursts. Repeated light stimulation (at second and third attempts) significantly decreased evoked burst latency in control and anisomycin-treated conditions, whereas K252a treatment did not influence the latency. *n* = 3 organoids. *P* = 0.0179; 0.0033 (2nd and 3rd, in comparison to 1st within control). **C** (i) Overlayed power histograms of evoked bursts and kernel density estimation (line) in the presence of K252a and anisomycin. Repeated light stimulation increased burst response complexity. (ii) Percentage of evoked bursts with and without secondary peaks. **D** Violin plots of burst response peak times. Red line indicates median. *n* = 50 bursts. **E** Representative crosstalk between the two connected organoids in self-evoked bursts. Color-coded maps (bottom circles) depict the voltage distribution from neurons in left and right cerebral organoids. *p < 0.05; one-way ANOVA followed by Tukey’s multiple-comparison test. Data are presented as mean values ± SD.

To gain insight into transmission of burst activity within connected organoids, we sorted and aligned the evoked burst activity (Fig. 7A). Closer examination revealed that optogenetic stimulations triggered multiple responses of neuronal activity within the evoked bursts. The latency of the bursts decreased with repeated stimulation in control (Fig. 7B), which was abolished by K252a. To dissect the evoked responses in further detail, we calculated probability histograms of the evoked bursts (Fig. 7C, D, Supplementary Fig. S11F). These revealed that the acute primary peaks are followed by secondary and tertiary responses. In the first stimulation, the primary peak was observed close to the end of the stimulation session, and weak secondary peak was observed. In the second and the third stimulation, the primary response peak became stronger, and temporally shifted towards beginning of the stimulation. The secondary and tertiary peaks also became prominent and stronger in the repeated stimulation sessions. The first response peak behaved similarly when the connected organoids were treated with K252a or anisomycin. The secondary/tertiary waves were also observed with K252a or Anisomycin treatment. Notably, two organoids often responded to light exposure with slightly shifted kinetics (Fig. 7E). We observed that the secondary and tertiary waves of the evoked bursts alternate between the two connected organoids (Fig. 7E). These results suggested that the activity in the connected organoids was generated and developed by a complex ensemble of activity within the circuit of the connected organoids, and the complexity can be observed as multiple peaks of the burst waveforms.

## Discussion

In this study, we demonstrated that connected cerebral organoids generate spontaneous oscillations that are more intense and complex than the single or fused organoids. The connected organoids responded to external stimuli and exhibited short-term plasticity. These demonstrated that the axonal connections could induce critical functionality of neuronal circuit tissue.

The intensity of neuronal activity was assessed as frequency of bursts and amplitude of LFP in various frequencies. They were both significantly increased by the inter-organoid connections with bundled axons. The spontaneous burst activity could be observed first in one organoid and followed by another with a slight delay, indicating that the two organoids can share activity through the axon bundle still with a small yet significant degree of functional separation as a neuronal circuit. Importantly, inhibition of activity at the axon bundles greatly reduced the burst frequency, and in turn, increased uncoordinated and unsynchronized isolated spike activity. These indicated that the connection could critically determine behavior of the whole neural circuit.

The connected organoids spontaneously produce complex activity patterns. We characterized the complexity of neuronal activity patterns with various methods, including LFPs, CV of burst intervals, and PAC modulation, all of which indicated that the connected organoids exhibit more complex activity than single or fused organoids. The complexity of neuronal activity patterns of the connected organoids demonstrates that the connection between organoids can change the network characteristics.

Interestingly, we also observed alteration of a population of cells in the connected organoids. The retrograde photo-labeling revealed that the neurons extending their axons into the inter-organoid bundle express higher levels of marker genes for neuronal activity and maturation. This highlights the relationship between the connection and the maturation, which occurs in certain populations of the cells in the connected organoids. This might be explained by the induction of cellular maturation through the action potentials transmitted more in the inter-organoid axons. It could also be possible that the relatively mature neurons extend their axons into the bundle and activate and further mature themselves through neuronal activity. Another potential reason for this is that pruning or cell death could be altered in the absence of neuronal input (from another organoid) in the single organoids.

The organoids extending their inter-organoid axons resemble the cortical neurons extending their axons into the cerebral tracts (“extrinsic pathway”). The axons in the tracts are structurally distinct from the axons going through the gray matter (“intrinsic pathway”). Interestingly, the human brain has many more intra-cortical “extrinsic pathway” axons in the white matter compared to most animals (e.g., rodents)^40^. This implies that the structural organization of the human brain could influence the property of neurons, potentially through activation and maturation of neurons that are connecting distant local circuits. To understand this process, the connected organoids can be used as a model to further investigate the mechanisms underlying the structure-induced alteration of the neuronal properties. This simplified organoids-on-a-chip approach could provide a way to understand the greater detail of the development and importance of the cerebral tracts in the human brain.

This study also provides evidence that the connected cerebral organoids have the capability to respond to external optical stimulation with short-term potentiation. The potentiation was observed in two ways: the sustained elevated frequency of burst activity, and the shortened induction time to respond to the stimuli. The first potentiation behavior was observed when the tissues generated from the 409B2 iPS line were stimulated extensively enough. When the stimulation time was short, the neurons did not exhibit potentiation and returned to the original activity pattern immediately. This indicates that the connected organoids changed their synaptic properties and adopted their frequency of burst during the extensive stimulation and changed their frequency of bursts. With another cell line 30HU-002, we did not observe sustained elevation of burst frequency. Instead, we observed that the train of stimulations led to depression of activity of the connected organoids when we stimulated one of the two organoids instead of the axon bundle. The response of the organoids to the axonal stimulations was less suppressive, indicating that the difference in location of stimulation altered the response of the organoids.

The shortened induction time was surprising, given that the stimulation was performed after the burst frequency returned to normal. This suggests that the plasticity of the connected organoid is not always appreciable as elevated frequency of bursts, but early induction of entrainment in response to a small number of stimulations. When a train of periodic stimulation is applied, effect of short-term plasticity is sustained and accumulated over the cycle of stimulations (1 s), and eventually the activity recruits a population of neurons large enough to cause a burst. Given the temporal property, the short-term plasticity could be synaptic augmentation or potentiation^41^. We speculate that metaplasticity for the short-term plasticity (synaptic augmentation and/or potentiation) is enhanced by the entraining stimulation in the first session, such that a few stimulations could induce the plastic changes before triggering entrained bursts through activation and recruitment of many neurons in the second and third sessions. This needs to be further analyzed and verified by a more detailed approach (e.g. patch clamp) in the future.

Currently, the major limitations of the connected organoids are insufficient maturation and lack of long-term potentiation. Human neurons, in general, develop and mature with longer time than neurons from other model animals, which limits the maturation of the neurons within reasonable time of culture and limits the capability of neurons to exhibit long-term potentiation. We also observed variable response and plasticity to external stimuli between the two iPS cell lines we tested in this study, although overall they show similar response. Optimization of the cells and tissues would be necessary to control variability to obtain more consistent response of the organoid and to evaluate small alterations of different types of plasticity (e.g. in disease models).

Further demonstrations such as connecting different types of neural organoids and/or connecting more than two organoids via axon bundles will establish novel avenues for understanding the human brain. On top of this, the addition of non-neuronal cell types and structures including glia cell and vasculature is necessary for a more sophisticated neural organoid model and maintaining for the long term (e.g., with perfusion culture). The model can be also used to model diseases that exhibit complex defects in brain activity (e.g., psychiatric disorders). Also, it would be useful to apply connected organoids to model diseases with structural defects (e.g. axonal tracts and corpus callosum) and examine structure-function relationship in the organoids. High density microelectrode array (HD-MEA) and calcium imaging has been used for analyzing network activity within organoids recently^41,42^, with which local network dynamics and circuitry within connected organoids can be further analyzed in future studies. The results of such experiments would not only allow us to utilize this tissue model in biology but also provide insight into the methods and principal mechanisms in information processing of the brain in the future.

## Methods

### Institutional approval

The use of human iPS cells was approved by the Institute of Industrial Science, The University of Tokyo. The human iPS cells were handled in accordance with approved protocols.

### PDMS chip fabrication

SU-8 master positive patterned molds were fabricated by standard photolithography techniques described elsewhere^43,44^. Briefly, SU-8 (2100 or 2075) was poured onto a silicon wafer (4 inches) and spin-coated (1200–1500 rpm, for 30 s). The wafer was baked for 9 min at 65 °C and for 40 min at 95 °C on a hotplate. Then, UV light (365 nm, 2.5–3.0 mWcm^2^) was irradiated with a photomask for 60–75 s. The wafer was baked for 7 min at 65 °C and for 13 min at 95 °C on a hotplate. After cooling down the wafer, SU-8 was developed by using SU-8 developer for 15 min, then washed with isopropyl alcohol (IPA) for three times. The wafer was baked for 3 min at 150 °C in the oven. The thickness of the SU-8 was approximately 150 µm.

Microfluidic device was made with a polydimethylsiloxane (PDMS) silicone elastomer kit Sylgard184 (Dow Corning). Silicone elastomer and a curing agent were mixed at a weight ratio of 10:1, degassed, poured onto the patterned SU-8 structures, and cured in the oven at 80 °C for 6 h. The holes for organoids and reference electrode were created with biopsy punches (1.5 mm and 2 mm, respectably). A glass ring (inner diameter: 22 mm, outer diameter: 25 mm) as the medium reservoir was glued to the PDMS device. The devices were sterilized by autoclave, 70% ethanol, and UV treatment.

### Human iPS cells

Human iPS cells were obtained from the Riken Cell Bank (409B2, HPS0076)^45^ or purchased (30HU-002, iXCells). The cells were maintained on ESC-qualified Matrigel-coated 6-well plates in mTeSR plus medium (STEMCELL Technologies) and subcultured every 5–7 days using ReLeSR reagent (STEMCELL Technologies).

### Cerebral organoids formation

To generate cerebral organoid, first, iPS cells were dissociated into single cells with TrypLE express. Then, 20, 000 cells were plated to each well of U-bottom ultra-low attachment 96 well plate (Prime surface, Sumitomo bakelite) in mTeSR plus with 10 μM of Y-23632. After 24 h, culture medium was replaced with neural induction medium (DMEM-F12, 15% (v/v) knockout serum replacement, 1% (v/v) MEM-NEAA, 1% (v/v) Glutamax, 100 nM LDN-193189, and 10 μM SB431542) and the medium was changed every 2 days. After 10 days of culture, the culture medium was replaced with 1:1 mixture of DMEM/F12 and Neurobasal medium supplemented with 0.5% (v/v) N2 supplement, 1% (v/v) B27 supplement without vitamin A, 1% (v/v) Glutamax, 0.5% (v/v) MEM-NEAA, 0.25 mg/ml (v/v) human insulin solution, and 1% (v/v) Penicillin/Streptomycin and the medium was changed every 2 days until 18 days. After 18 days of culture, the culture medium was replaced with maintenance medium (Neurobasal medium supplemented with 0.5% (v/v) N2 supplement, 1% (v/v) B27 supplement with vitamin A, 1% (v/v) Glutamax, 0.5% (v/v) MEM-NEAA, 0.25 mg/ml (v/v) human insulin solution, 20 ng/ml BDNF, and 200 mM ascorbic acid, and 1% (v/v) Penicillin/Streptomycin). Cerebral organoids were cultured for four weeks and subjected to the connected organoid formation.

### Connected organoids formation in the culture chip

Two cerebral organoids were connected in the microfluidic device. The previously reported protocol has been modified^28^. Briefly, the PDMS devices were bonded to MEA probes (MED-R5004A-NR). We aligned the PDMS device with the electrodes during the bonding. The PDMS chip was applied on the MEA probe with residual 70% ethanol used for disinfection. As the ethanol dries, the PDMS chip attaches to the MEA probe. The microchannel was coated with Matrigel (Corning) in DMEM/F12 (1:30) for 1 h at RT. The coating solution was replaced with maintenance medium (Neurobasal medium supplemented with 2% (v/v) B27 supplement with vitamin A, 1% (v/v) Glutamax, 20 ng/ml BDNF, and 1% (v/v) Penicillin/Streptomycin). Cerebral organoids were then placed into the holes and settled down to the bottom by gravity. The maintenance medium was replaced every 2 days.

### Multi-electrode array measurement and post-analysis

To capture neuronal activity with multi-electrode array, maintenance medium was replaced with Brainphys without phenol red supplemented with 2% (v/v) B27 supplement with vitamin A, 1% (v/v) Glutamax, 20 ng/ml BDNF, and 1% (v/v) Penicillin/Streptomycin before measurement. The PDMS-MEA chip was set to the MED64 system (Alpha MED Scientific) and electrical signals from all 64 electrodes were recorded for 5–30 min at 37 °C at 20,000 Hz sampling rate. The recording noise was eliminated by band-pass filter between 0.1–10000 Hz during the measurement.

The raw signal was further fileted by a bandpass filter (300–3000 Hz) for spike analysis. A spike was counted when the extracellularly recorded signal exceeded a threshold of ±5σ, where σ represents the standard deviation of the baseline noise during quiescent periods. A burst was counted when five spikes were observed within 100 msec window on the electrode. Low pass filter (<1000 Hz) was applied to the raw data for local field potential (LFP) analysis. All the analysis and calculation including wavelet transformation, phase-amplitude coupling, and neuronal avalanches were conducted using MATLAB software.

### Wavelet coherence and cross-correlation

Wavelet coherence is a measure of correlation between two signals at specific frequency. To decompose the neural signals into distinct frequency bands, we employed Continuous Wavelet Transform (CWT). Wavelet coherence from LFP recording, f(t) was calculated using function cwt() and icwt() in package “Wavelet Toolbox” of MATLAB. Cross-correlation was calculated with xcorr() function in MATLAB. We used scaleopt option to normalize the cross-correlation as 1 when two signals had no time lag.

### Phase-amplitude coupling analysis

We conducted a phase-amplitude coupling (PAC) analysis to investigate the interactions between different neural oscillatory frequency bands as previously described^21^. The analysis was performed on low-pass filtered neural signals obtained from specific electrodes. To decompose the neural signals into distinct frequency bands, we employed Continuous Wavelet Transform (CWT) which allowed us to obtain time-frequency representations of the signals, preserving both temporal and spectral information. The CWT was applied to the signal, yielding wavelet coefficients across a range of frequencies. Using the inverse CWT, we isolated specific frequency bands pertinent to our study: delta (0.5–4 Hz), theta (4–8 Hz), and gamma (30–300 Hz). We then applied the Hilbert transform to the reconstructed signals of each frequency band. This transformation provided us with the analytic signal, from which we extracted the instantaneous phase and amplitude. We calculated the modulation index (MI) to quantify the degree of coupling between the phase of one frequency band and the amplitude of another. The phase data was divided into bins spanning from -π to π. We used 36 bins (10 degrees per bin). Amplitudes were binned according to their corresponding phase values. The mean amplitude was calculated for each bin and normalized. We computed the Kullback-Leibler (KL) distance, which measures the divergence of the observed amplitude distribution from a uniform distribution. The MI was derived from the KL distance, normalized by the logarithm of the number of bins, providing a measure of phase-amplitude coupling strength.

### Neuronal avalanches

Neuronal avalanches were characterized by the continuous activity patterns within the organoids. The calculating time bin (Δt) was set as 3 msec. We combined signals detected from all electrodes, and grouped multiple consecutive signals that were separated no more than 3 msec from at least another signal, which was counted as a neuronal avalanche. The length of the train of signals were measured for each avalanche, and the histogram was plotted.

The probability of avalanches was calculated by the following equation to obtain *α*, the exponent of power law.

1 $$P\left(S\right)=k{S}^{-\alpha }$$

*P*(*S*) is the probability of observing an avalanche of size *S*, giving the slope of relationship in a log-log coordinates, and *k* is a proportionality.

### Optogenetic manipulation of the connected organoids

To manipulate the activities of the connected organoids, we employed optogenetic tools (Supplementary Fig. S2). AAV-CAG-hChR2^H134R^-tdTomato was a gift from Karel Svoboda (Addgene plasmid # 28017). pAAV-CAG-ArchT-GFP was a gift from Edward Boyden (Addgene plasmid # 29777). Briefly, 5 µL of AAV virus prep were mixed with 500 µL of maintenance medium and then the mixture was replaced with the medium in the microfluidic device before at least 72 h prior to the MEA measurement. Fiber-coupled LED of 470 nm (M470F3 − 470 nm, 17.2 mW (Min) Fiber-Coupled LED, 1000 mA, Thorlabs) for hChR2^H134R^ and 565 nm LED (M565F3, 565 nm, 9.9 mW (Min) Fiber-Coupled LED, 700 mA, Thorlabs) for Arch-T were controlled by T-cube LED driver (LEDD1B, 1.2 A, Thorlabs). Light was delivered to microfluidic device through multimode fiber (0.22 NA, High-OH, Ø105 µm Core, 250–1200 nm, Thorlabs). TTL pulse was generated by Arduino to control the LED driver. For drug treatments in potentiation experiments, K252a (abcam) and Anisomycin (Wako) was administered to the cell culture at least 1 h before measurement at a final concentration of 25 nM and 5 µM, respectively.

### Determination of axon-bundle associated neurons by photo-convertible fluorescent Kaede

To identify axon-bundle associated neurons in the connected organoids, photo-convertible fluorescent Kaede were transfected to connected organoid. First, AAV-CAG-Kaede plasmid was constructed (Fig. 3A). AAV was produced in AAVpro 293T (Takara) and purified with AAVpro Purification kit midi (Takara) according to the manufacturer’s protocol. The connected organoids were infected with the AAV at 4–6 weeks, then subjected to photo-conversion experiment at 7 weeks of culture. To visualize axon-bundle associated neurons in the connected organoids, 405 nm light was irradiated to the region of axon bundles with a Nikon confocal microscope (1.5 mm ×0.5 mm ×0.2 mm, 5×, total time: 60 min, Nikon A1R). Then, we waited for three hours in total to allow the photo-converted Kaede-red to trafficked to the cell bodies. The organoids were dissociated by Accumax for 10–30 min at 37 °C and centrifuged at 200 × *g* for 5 min. For flow cytometry, the dissociated cells were resuspended with PBS containing 1% BSA. Kaede red-positive/Kaede green-negative populations and Kaede red-negative/Kaede green-positive populations were collected as axon-bundle-associated neurons and non-axon-bundle-associated neurons, respectively, using BD FACS melody. After sorting, total RNA was collected for RT-PCR analysis.

### Immunostaining

Organoids were fixed in 4% paraformaldehyde (PFA) and 4% sucrose at 4 °C for 24 h, washed 3 times with PBS (10 min incubation at RT for each wash), and transferred to 30% sucrose solution for incubation overnight at 4 °C. Organoids were then embedded in O.C.T compound and frozen on a liquid nitrogen-cooled metal box. Organoids were then subjected to cryosectioning to obtain 20-µm-thick slices.

Cells were fixed with 4% paraformaldehyde for 20 min and then permeabilized with 0.2% Triton X-100 for 5 min. After blocking with 1% bovine serum albumin (BSA) for 2 h, the cells were incubated for 2 h at room temperature or 4 degrees overnight with a primary antibody. A secondary antibody was then administered for up to 2 h at room temperature. The primary antibodies were mouse anti-neuron-specific βIII tubulin (Biolegend, 801202, 1:1200), rabbit anti-neuron-specific βIII tubulin (Sigma ZooMAb, ZRB1140, 1:200), rabbit anti-PAX6 (Wako, 015-27293, 1:500), mouse anti-human GAD67 (Santa Cruz, sc-28376, 1:100), rabbit anti-human VGLUT1 (Sigma ZooMAb, ZRB2374, 1:200), rat anti-human CTIP2 (Abcam, ab18465 1:200), mouse anti-Tau1 (Merck, MAB3420, 1:1000), and rabbit anti-synapsin (Merck, AB1543, 1:500). The secondary antibodies were Alexa Fluor 568 goat anti-rabbit IgG (H + L) (Thermo Fisher Scientific, A11036), Alexa Fluor 488 goat anti-mouse IgG (H + L) (Thermo Fisher Scientific, A11029), and Alexa Fluor 488 goat anti-rat IgG H&L (Abcam, ab150165). Nuclei were stained with Hoechst dye for 20 min at room temperature. All cells and samples were observed using a fluorescent microscope (Axio Observer, Zeiss) or a confocal laser scanning microscope (BioPipeline LIVE, A1R, Nikon).

### Reverse-transcription PCR

To measure the biological activity of the cerebral organoids, total RNA was isolated from tissues with TriPure (Sigma) from the tissues. Reverse transcription was performed using a KOD One (Toyobo). The primer sequences are shown in Supplementary Table S1. RT-PCR was performed with CFX Connect (BioRad) using KAPA SYBR FAST qPCR Master Mix (KAPA Biosystems). The mRNA level of glyceraldehyde 3-phosphate dehydrogenase (GAPDH) was used as the internal standard in all experiments. The RT-PCR experiments were repeated at least three times with cDNAs prepared from separate tissues.

### Statistics & reproducibility

The reported values are the means of a minimum of three independent experiments. Data are presented as the mean ± SD. Comparisons were performed using one-way analysis of variance (ANOVA), with post hoc pairwise comparisons carried out using the Tukey–Kramer method unless otherwise stated. Statistical tests were performed using GraphPad Prism, MATLAB or R studio. We excluded organoids that had no electrical activity recorded.

### Reporting summary

Further information on research design is available in the Nature Portfolio Reporting Summary linked to this article.

### Supplementary information

Supplementary Information

Reporting Summary

### Source data

Source Data

## Data Availability

All data that support the findings of this paper are presented within the paper and the Supplementary Information. scRNA-seq data (GEO:GSE190729) used in this study has been deposited to NCBI. Additional data related to this paper may be requested from the authors. Source data are provided with this paper.

## Code availability

Source codes for MEA analysis and used for in silico organoid model are available at Mendeley Data (10.17632/vcf5f2k5yb.1), and github (https://github.com/Ceramic-Blue-Tim/in-silico-connected-organoid-model; https://github.com/utokyoIkeuchilab/complex_activity_data_analysis).
