# Supplementary material for: Complex activity and short-term plasticity of human cerebral organoids reciprocally connected with axons
Source: Nat Commun. 2024 Apr 10;15:2945. doi: 10.1038/s41467-024-46787-7 (PMC11006899; doi:10.1038/s41467-024-46787-7)
Supplement: Supplementary file 1 — Supplementary Information [file 41467_2024_46787_MOESM1_ESM.pdf]

Complex Activity and Short-Term Plasticity of Human Cerebral Organoids Reciprocally Connected with Axons

This PDF file includes:

Supplementary Methods
Figs. S1 to S13
Tables S1 to S2
Supplementary reference

Supplementary Methods

In silico model of connected organoids

In silico simulations of neuronal activity in connected organoids were performed based on Hodgkin-Huxley formalism[1] running on MATLAB. Briefly, two types of cortical neurons, regular spiking neurons (RS) and fast spiking neurons (FS)[2], were used as excitatory neurons and inhibitory neurons respectively. Please refer to the previous work for a detailed description of the mathematical implementation and parameters[2]. As creating simplest model, one single organoid structure consists in 90 RS and 10 FS (100 neurons in a total), and two types of neurons were randomly distributed to the circle shape (diameter: 138.25 μm). RS ad FS were connected using AMPA and GABA synapses[3]. All synapses inside an organoid share the same synaptic weight (0.05) and connection probability (0.10). In addition to intra-organoid connection, in fused organoid and connected organoid model, two organoids were connected as inter-organoid connection with a different synaptic weight (0.1) and probability (0.05). In fused organoids, synaptic connection probability between organoids was proportional to the distance between 2 neurons, promoting connection for neurons on the edge facing the opposite organoid. In connected organoid, synaptic connection probability was determined by the position of each neuron to its belonging organoid to maximize connection for neurons on the outside of the organoid. Spontaneous activity was tuned through the synaptic noise parameters[4] following an Ornstein-Uhlenbeck process. Spikes were detected above a certain threshold (0 mV).

Whole cell patch clamp recordings

To characterize the electrophysiological property of neurons in connected organoid after 8.5 weeks from differentiation, whole-cell patch-clamp recording was performed. Connected organoids were formed in thin PDMS device (< 1 mm) without MEA probe so that the patch pipettes can access the neurons in the connected organoids (Fig. S2A). Whole-cell patch-clamp recordings were obtained from individual cells with glass pipettes (4–9 MΩ) filled with internal solution containing 130 mM potassium gluconate, 8 mM KCl, 1 mM $MgCl_2$, 0.6 mM EGTA, 10 mM HEPES, 3 mM Na_2ATP, 0.5 mM Na_2GTP, 10 mM Tris–phosphocreatine, and 0.2% biocytin (with pH adjusted to 7.35 with KOH). During recordings, warm brainphys medium was continuously perfused into the culture dish at the rate of 2.0 ml/min to maintain the organoid at 37°C. Recordings were performed using MultiClamp700B amplifiers (Molecular Devices), filtered at 3 kHz using a Bessel filter and digitized at 20 kHz with Digidata 1550B digitizer (Molecular Devices) with pClamp software. In order to characterize membrane potential change responded by the injection of current, hyperpolarizing and depolarizing square wave current pulses were injected with target neurons (−10 to +20 pA, +2 pA increment, 1 s, Fig. S2C, D). Also, spontaneous action potential was recorded (Fig. S2E). 13 individual neurons from two connected organoids in total were recorded and 6 neurons were further analyzed for current injection. All recording data sets were shown in the table (Fig. S2B). The quantification was implemented using the MATLAB and Origin Pro. After the recordings, connected organoids were fixed and permeabilized with 4% paraformaldehyde and 0.2% TritonX-100, and subsequently stained with Alexa Fluor 647 Streptavidin (Thermo Fisher, S21374, 1:500) to detect biocytin to visualize the recorded neurons.

scRNA-seq and data processing

Single, fused, and connected organoid at 7 weeks of culture on PDMS-MEA chip were first extracted

from PDMS device in warmed maintenance medium and centrifuged at 100 xg for 30 seconds. To get single-cell suspensions, the organoids were dissociated by AccuMax for 10-30 min at 37°C and then centrifuged 200 xg for 5 min. Then, the pelleted cells were re-suspended in DMEM containing 10% FBS to be subjected to 10x Genomics Chromium single-cell RNA-seq library preparation, according to the manufacturer's protocol. Finally, the library was sequencing with 150-bp paired-end reads on DNBSEQ. The sequencing data were processed using Cell Ranger analysis pipeline v3 with default parameters. Reads were aligned to human reference genome (GRCh38). Cell Ranger output "filtered gene-barcoded" count matrix was loaded into Scanpy with other python packages (scanpy==1.8.1 anndata==0.7.6 umap==0.5.1 numpy==1.19.5 scipy==1.4.1 pandas==1.1.5 scikit-learn==0.22.2.post1 statsmodels==0.10.2 python-igraph==0.9.6 pynndescent==0.5.4) for downstream analysis[5]. Poor-quality cells were excluded based on the following criterion: min_genes > 200 and min_cells < 3, mitochondrial gene percentage < 10%, and nFeatures <4000. Cells with percentage of hemoglobin reads > 5% were discarded. In total, 17,636 cells remained for subsequent analysis. Batch effect correction was performed.

To reduce the dimension of data, principal component analysis (PCA) was performed with Scanpy. UMAP was employed for visualization of the clustered data.

Simultaneous Ca^{2+} imaging with customized microscopes

To visualize neuronal activity by fluorescent, Ca^{2+} indicator (GCaMP6f driven by CAG promoter) were transfected by using AAV1. pAAV.CAG.GCaMP6f.WPRE.SV40 which was a gift from Douglas Kim & GENIE Project (Addgene #100836). Briefly, the AAV was added to the medium of the connected organoids in the device before at least 3 days of measurement. The organoids were exposed to the AAV for 6-12 hours. Maintenance medium was replaced with BrainPhys supplemented with 2% (v/v) B27 supplement with vitamin A, 1% (v/v) GlutaMax, 20 ng/ml BDNF, and 1%(v/v) Penicillin/Streptomycin before 30 min of measurement. The configuration of the microscopes was described in Fig. S10. The connected organoids were too far to be imaged by a single microscope with high resolution, but too close to be imaged with two inverted microscopes. We combined an inverted microscope with an upright microscope, whose optical axes were slightly shifted to image organoids from top or bottom independently. The brightfield lamp and condenser were removed from the inverted microscope to accommodate the upright microscope integration. The relative position of the microscopes can be shifted with a stage under the inverted microscope. The upright microscope was also used in combination with electrical recordings with MED64. Time-lapse images were captured at least at a frame rate of 20 fps for 10 min using ORCA Flash (Hamamatsu) or NF300 (Wraymer). Data analysis was carried out using MATLAB (MathWorks). Regions of interest (ROIs) were manually drawn around the cell body of neurons in organoids. For each ROI time series, baseline fluorescence was defined as the average of the lowest 10% of samples. $\Delta F/F$ was computed as $(F - F0)/F0 \times 100$, where F is the instantaneous fluorescence from the raw ROI time series.

Knock-in for GFP, mCherry, and Brainbow human iPS cells

To visualize axonal outgrowth and neurons in organoid with multiple fluorescence, GFP, mCherry, or Brainbow fluorescent protein were stably expressed in the human iPS cells. GFP, mCherry, or Brainbow sequence was inserted to the AAVS1 safe harbor locus. 5 µg of PX458-AAVS1 plasmid and 5 µg of AAVS1-Pur-CAG-EGFP plasmid[6] or 5 µg of AAVS1-Pur-CAG-mCherry plasmid [6] or AAVS1-Pur-CAG-Brainbow TEC were mixed with 1×10^6 human iPS cells in 100 µL of Opti-MEM. The plasmid-cell mixture was transferred to a NEPA cuvette (EC-002S) and electroporated by a NEPA21 electroporator (Poring plus: voltage:125V, pulse length: 5 msec, pulse: 50 msec; number of pulses: 2, decay rate 10%. Transfer pulse: voltage: 20V, pulse length: 50 msec, pulse:

50 msec; number of pulses: 2, decay rate 40%). The electroporated iPS cells were seeded onto Matrigel-coated plate in mTeSR plus with 10 μM of Y-23632. After 24 h, transfected iPS cells were selected by 0.75 μg/ml puromycin treatment for 2 days. The cells were then expanded. PX458-AAVS1, AAVS1-Pur-CAG-EGFP, and AAVS1-Pur-CAG-mCherry plasmids were gifts from Drs. Adam Karpf and Su-Chun Zhang (Addgene #113194, #80945, and #80946). AAVS1-Pur-CAG-Brainbow plasmid was constructed based on AAVS1-P-CAG-DEST (Addgene #80490) and pME-Brainbow TEC (Addgene #82405)

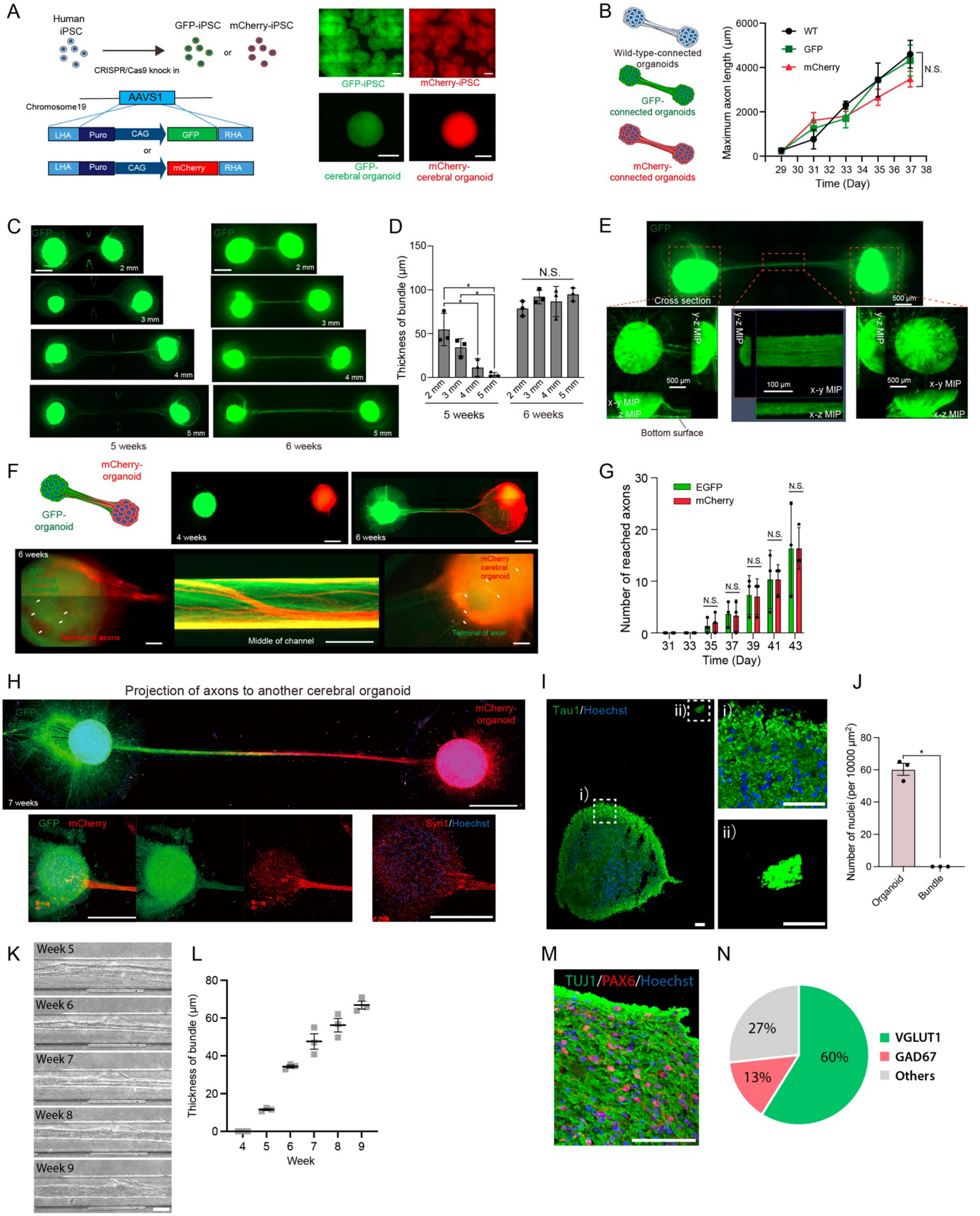
A
Human iPSC
CRISPR/Cas9 knock in
GFP-iPSC
or
mCherry-iPSC
AAVS1
Chromosome19
LHA
Puro
CAG
GFP
RHA
or
LHA
Puro
CAG
mCherry
RHA
GFP-iPSC
mCherry-iPSC
GFP-cerebral organoid
mCherry-cerebral organoid
B
Wild-type-connected organoids
GFP-connected organoids
mCherry-connected organoids
Maximum axon length (μm)
WT
GFP
mCherry
N.S.
Time (Day)
C
5 weeks
6 weeks
D
Thickness of bundle (μm)
N.S.
5 weeks
6 weeks
E
Cross section
Bottom surface
F
GFP-organoid
mCherry-organoid
4 weeks
6 weeks
Terminal of axons
Middle of channel
Terminal of axon
G
Number of reached axons
EGFP
mCherry
Time (Day)
H
Projection of axons to another cerebral organoid
mCherry-organoid
7 weeks
GFP
mCherry
Syn1/Hoechst
I
Tau1/Hoechst
J
Number of nuclei (per 10000 μm²)
Organoid
Bundle
K
Week 5
Week 6
Week 7
Week 8
Week 9
L
Thickness of bundle (μm)
Week
M
TUJ1/PAX6/Hoechst
N
VGLUT1
GAD67
Others
60%
13%
27%

Fig. S1 | Visualization of projected axons by CRISPR knock-in cerebral organoids.

(**A**) CRISPR Knock-in of CAG promoter-driven GFP and mCherry fluorescent tags into the AAVS1 safe harbor locus in human iPS cells. GFP- or mCherry-labeled cerebral organoids were generated to visualize axon outgrowth in PDMS chips. Scale bar: 500 µm. (**B**) No significant phenotypic differences between wild-type and GFP- or mCherry-labeled cerebral organoids in terms of bundle formation speed. n = 3 organoids. (**C**) Tracking of axonal outgrowth of GFP-labeled connected cerebral organoids in different lengths of axon bundle (2, 3, 4, and 5 mm) at 5 and 6 weeks. Scale bar: 500 µm. (**D**) The thickness of axon bundles of GFP-labeled connected organoids at the center of microchannel. n = 3 organoids. P = 0.0084 (4mm); 0.0029 and 0.0453 (5mm) (**E**) 3D confocal microscopic images of GFP-labeled connected organoid. (**F**) GFP-labeled and mCherry-labeled cerebral organoids were connected. GFP- and mCherry-labeled axons projected to another cerebral organoid via merged thick axon bundle after two weeks in the chip. Scale bar: 500 µm (top panels); 100 µm (bottom panels). (**G**) Number of axons reached the other organoid. n = 3 organoids. (**H**) Projection of axons to another cerebral organoid. Immunostaining of SynI revealed synapse connections between organoids. Scale bar: 500 µm. **(I)** Representative image of Tau1 and Hoechst staining of **(i)** an organoid and **(ii)** axon bundle region from a connected organoid. Scale bar: 50 µm. **(J)** Quantification of number of nuclei in organoid and axon bundle regions of connected organoids. No cell bodies were observed in axon bundles. n = 3. P = 0.0038. **(K)** Representative time course images of axon bundle formation of a connected organoid generated from 30HU-002. n = 3. Scale bar: 100 µm. **(L)** Quantification of axon bundle thickness. n = 3 organoids. **(M)** Immunostaining of an organoid with TUJ1 and PAX6 after 8 weeks of culture. Scale bar: 100 µm. **(N)** The proportions of excitatory neurons, inhibitory neurons, and other neurons in the organoids. n = 3. *$p<0.05$; one-way ANOVA with Tukey's multiple comparison test for 1D, and student's t-test (two-sided) for 1G and J. Data are presented as mean values ± SD.

A

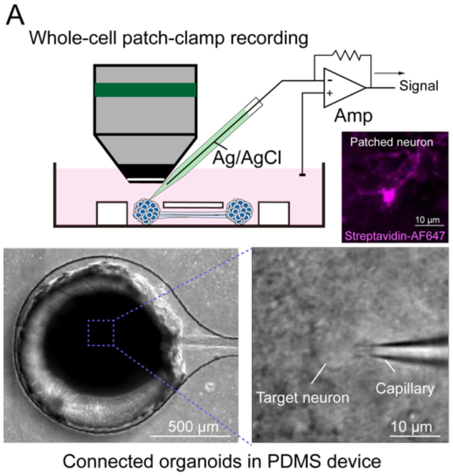

B

Neuron #	Depth (µm)	Vrest (mV)	Rinput (Mohm)	Cmembrane (pF)	AP threshold (mV)	AP amplitude (mV)	AP half width (ms)	Classified label
#1	0	-30.4	975.4	13.6				MF
#2	15	-31.8	624.5	20.8	-37.6	26.3	11.8	Unsustained
#3	20	-25.2	1525.9	13.8				MF
#4	20	-28.5	317.7	40.6	-37.8	33.5	9.1	Unsustained
#5	25	-25.1	1411.5	20.3				MF
#6	25	-22.6	1161.2	23.7				NF
#7	25	-20.2	299.3	35.1				MF
#8	30	-48.4	750.0	60.1	-34.2	57.5	2.15	Sustained
#9	30	-40.1	287.2	38.4	-46.5	5.9	9.1	Unsustained
#10	40	-42.8	624.3	46.5	-34.2	46.9	2.55	Sustained
#11	40	-29.9	235.7	25.2				NF
#12	40	-20.9	287.3	24.1				NF
#13	55	-24.7	1093.0	37.6	-34.1	40.4	2.7	Unsustained

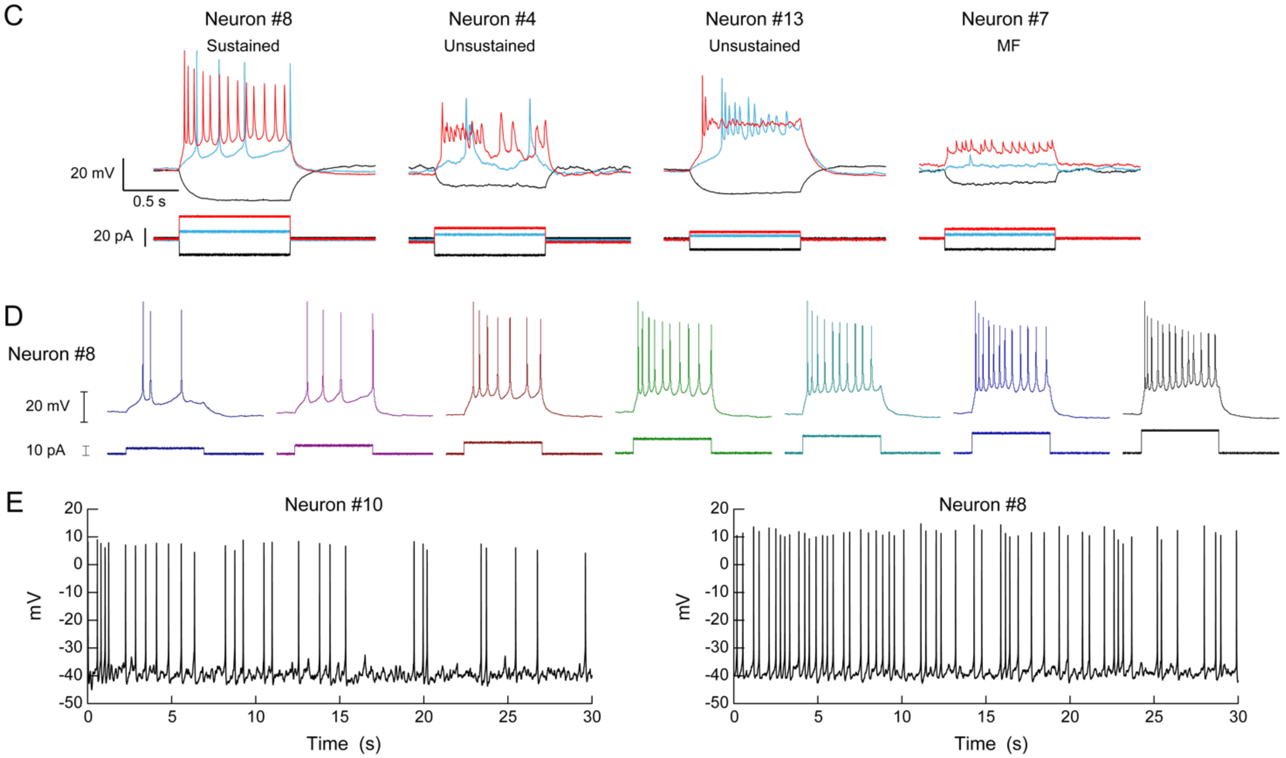

Fig. S2 | Electrophysiological properties of neurons in the connected organoids.

(**A**) Setup for whole-cell patch-clamp recording. Neuronal activities were recorded from the neurons in connected organoid in PDMS device. (**B**) All recording data set sorted by the depth including the depth from the surface of organoids in target neurons, resting membrane potential (V_{rest}), input resistance (R_{input}), membrane capacitance ($C_{membrane}$), action potential (AP) threshold, AP amplitude, AP half width, and classified label. MF; miniature firing. NF; no firing. (**C**) Membrane potential in five representative neurons responded with the injection of current. (**D**) Concatenate plot of membrane potential in a representative single neuron of sustained firing (#8) injected with currents from +6 pA to +18 pA. (**E**) Spontaneous AP in two representative neurons (#10 and #8).

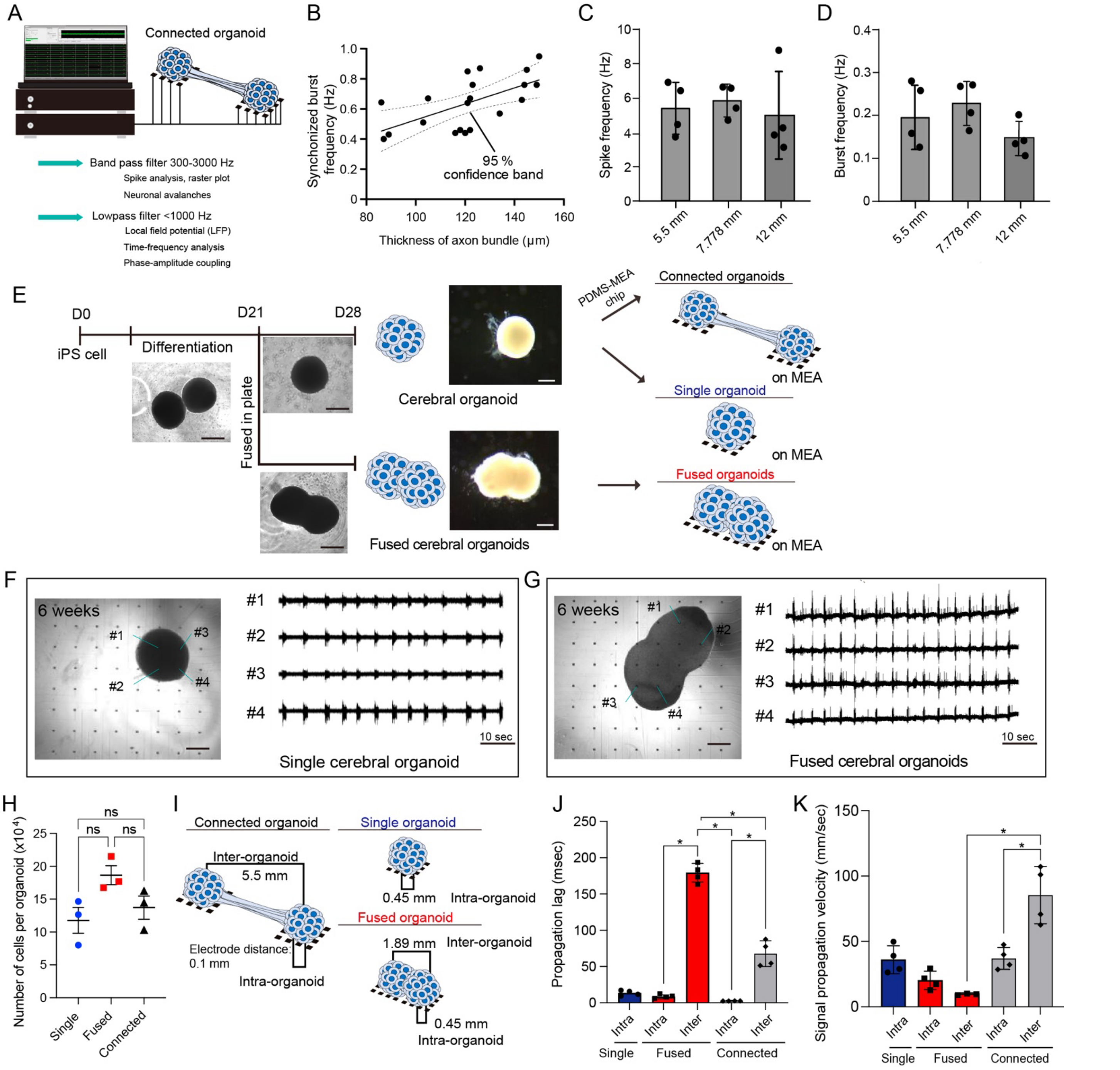

Fig. S3 | Comparison of single, fused, and connected organoids.

(A) Schematic illustration of generating single, fused, and connected organoids. Scale bar: 500 µm. **(B)** Correlation between thickness of axon bundle and synchronized burst frequency. Burst frequency increased with increasing the axon bundle thickness. **(C and D)** Spike **(C)** and burst **(D)** frequency of connected organoids with varying axon bundle length. No significant difference was found. n = 4 organoids. **(E)** All cerebral organoids were generated in the same manner for up to 21 days. To generate fused organoids, two cerebral organoids were placed into a well of low-adherent 96-well plate. Single and fused organoids were then placed onto MEA probes on day 28. Organoids were introduced into a microfluidic chip for generating connected organoid on day 28. After 2 weeks of culture on MEA probes, neuronal activities were measured. Scale bar: 500 µm. **(F)** A representative image of a single organoid on a MEA probe. Periodic and synchronized neuronal activity was observed. Scale bar: 500 µm. **(G)** A representative image of fused organoids on an MEA probe. More aggressive periodic and

synchronized neuronal activity than single organoid was observed. Scale bar: 500 µm. **(H)** Single, fused or connected organoids were dissociated to count the number of cells. No significant difference in cell number was found. n = 4 organoids. P = 0.0701 (single/fused); 0.1720 (single/connected); 0.1920 (fused/connected). **(I)** Illustration of intra- and inter-organoid activity lag detection by electrodes. **(J)** Quantification of propagation lag between electrodes. n = 4 organoids. 'Inter-connected' exhibited significantly smaller lag than 'inter-fused'. P = 1.4e-12 (fused intra/inter); 8.0e-10 (fused/connected inter);1.3e-6 (connected intra/inter). **(K)** Signal propagation velocity. 'Inter-connected' signal transduction was faster than others. n = 4 organoids. P = 1.3e-5 (fused/connected); 0.0006 (connected). *$p<0.05$; one-way ANOVA with Tukey's multiple comparison test. Data are presented as mean values ± SD.

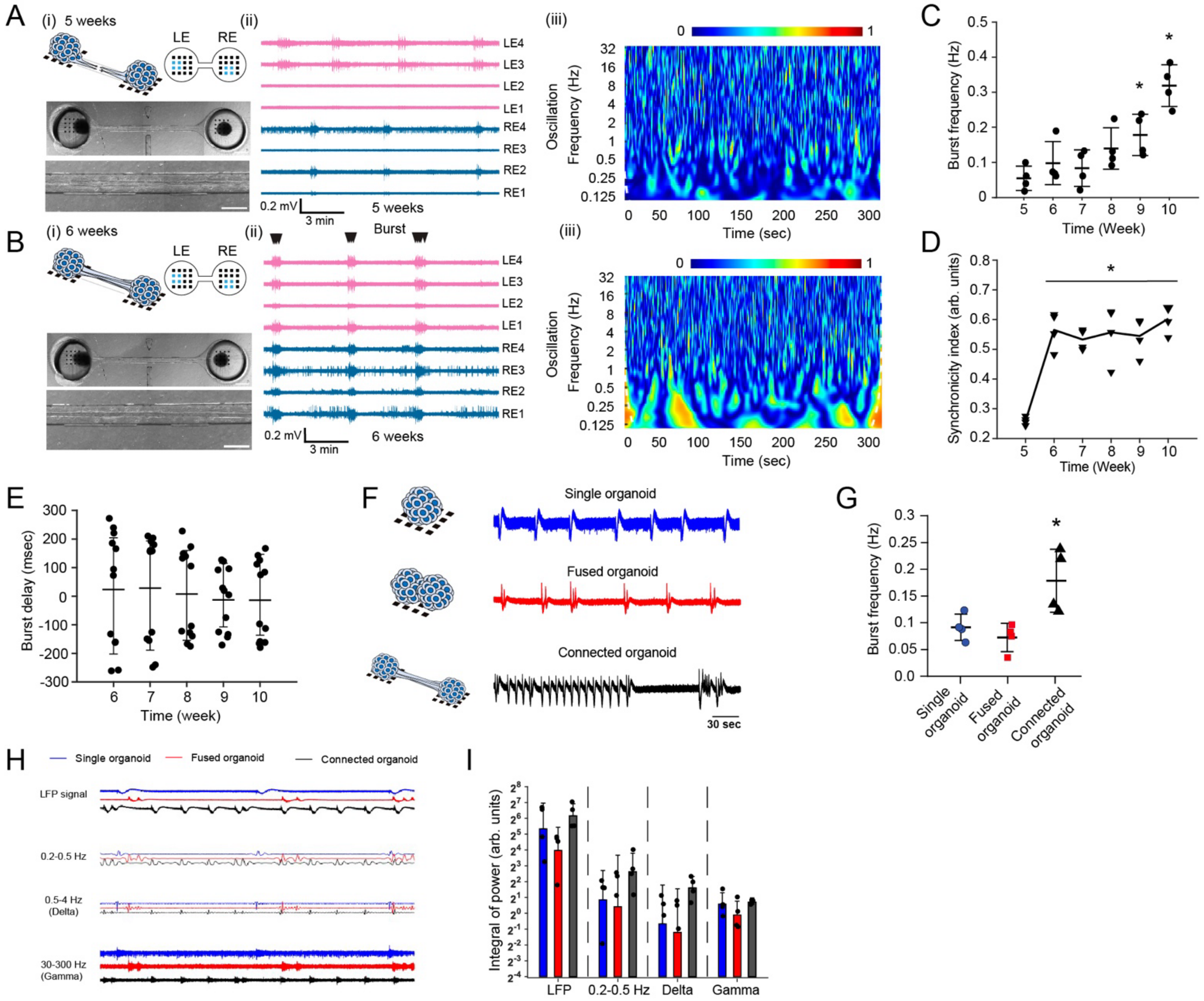

Fig. S4 | Activity of connected organoids generated from 30HU-002.

(A) (i) Representative images of connected organoids after 5 weeks of culture. Scale bar: 150 µm. (iii) Filtered signals from four representative electrodes under each organoid. (iii) Wavelet coherence between signals from an electrode under one organoid and another electrode under the other connected organoid. **(B)** (i) Representative images, (ii) filtered signals, and (iii) wavelet coherence of the connected organoids after 6 weeks of culture are shown as in A. Black arrowheads in (ii) represent synchronized burst activities associated with dense spikes. **(C)** Burst frequency increased significantly with culture time. n = 4 organoids. P = 0.0498; 3.0031e-5. **(D)** Synchronicity of activity in the two connected organoids increased during the culture period. n =4. P = 9.6e-6; 4.2e-5; 1.3e-5; 2.4e-5; 1.9e-6 (weeks 6-10, relative to week 5). **(E)** Burst delay after different culture periods. n = 12 bursts. **(F)** Representative neuronal activity of the single, fused and connected organoids. **(G)** Burst frequency of the single, fused and connected organoids. n = 4 organoids. P = 0.0317. **(H and I)** Inverse continuous wavelet transformation in the 0.2–0.5-Hz, 0.5–4-Hz (delta), and 30–300-Hz (gamma) bands. n = 4 organoids. *p<0.05; one-way ANOVA with Tukey's multiple comparison test. Data are presented as mean values ± SD.

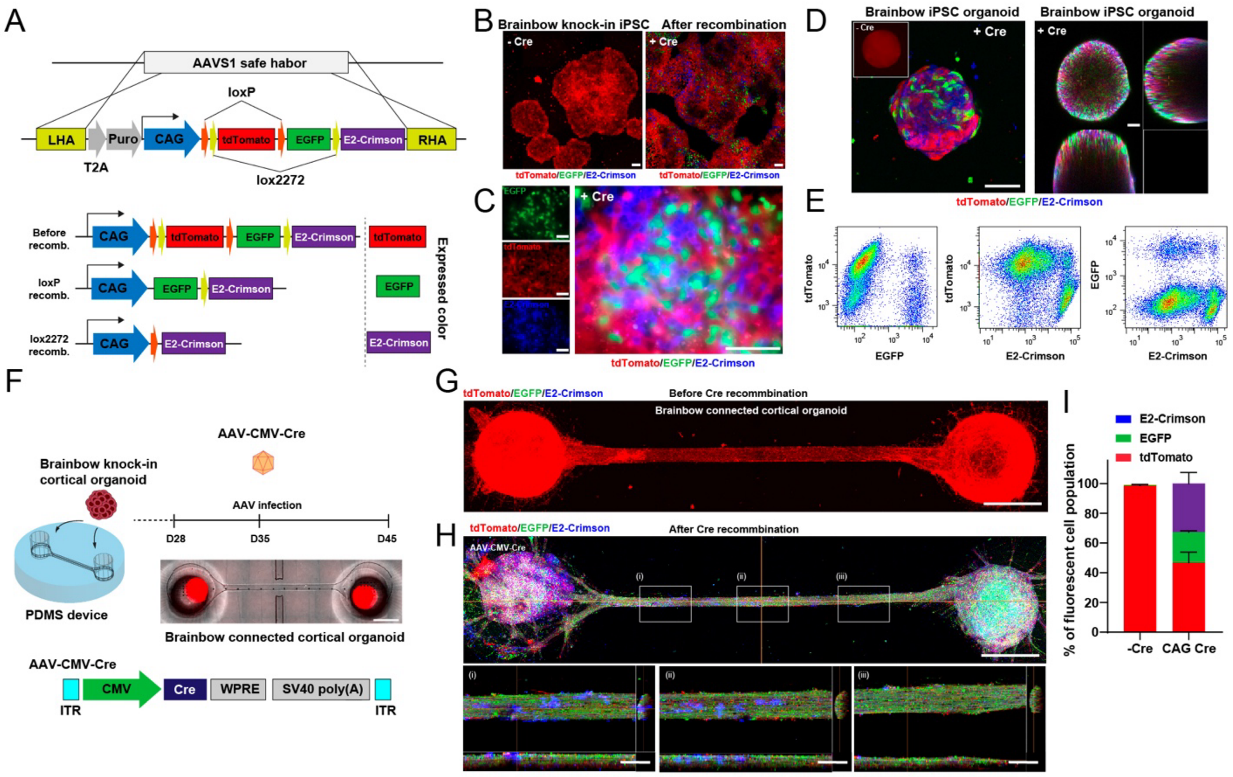

Fig. S5 | Visualization of neuronal morphology within the connected organoids with Brainbow.

(A) Brainbow constructs, which express tdTomato, EGFP, and E2 crimson under CAG promotor were integrated into the AAVS1 locus by CRISPR knock-in. In the presence of Cre recombinase, Cre-lox recombination can be induced, and tdTomato, EGFP, E2 crimson were randomly selected to be expressed in the neuron. **(B)** Immunostaining of Brainbow knock-in iPS cells with or without Cre recombinase. Scale bar: 50 µm. **(C)** In the presence of Cre recombinase, tdTomato, EGFP, E2 crimson in iPSC is successfully expressed. Scale bar: 100 µm. **(D)** Brainbow-cerebral organoids in the presence or absence of Cre recombinase. Scale bar: 100 µm. **(E)** Flow cytometry followed by dissociation of organoids confirmed the expression of three types of fluorescent proteins. **(F)** Schematic procedure of formation of Brainbow-connected organoids and virus infection (AAV-CMV-Cre). Brainbow cerebral organoids were introduced into the microfluidic device on day 28 from the differentiation. Then, AAV was added on day 35. **(G)** Fluorescent images of the Brainbow connected organoids before AAV treatment. Scale bar: 1 mm **(H)** Fluorescent images of the Brainbow connected organoids after AAV treatment on day 45. The AAV successfully induced Cre-lox recombination and fluorescent color changes. Scale bar: 1 mm (top); 150 µm (i-iii). **(I)** Expression of three fluorescent proteins were measured in the connected organoids by flow cytometry. n = 3. Data are presented as mean values ± SD.

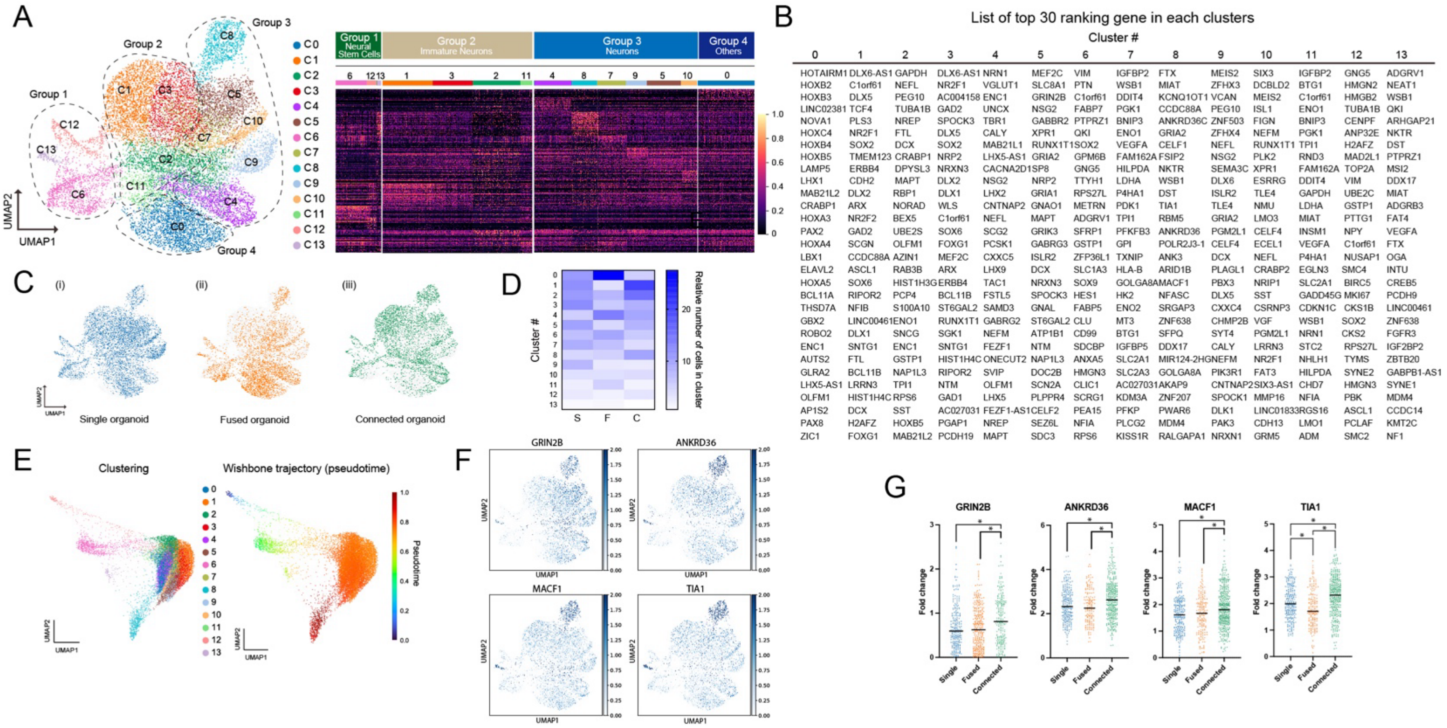

Fig. S6 | Single cell RNA-seq of single, fused and connected organoids.

(A) UMAP plot of 17,636 single cells with Leiden clustering implemented with single, fused, and connected organoids. All the cells were classified as 14 clusters. The 14 clusters were categorized into four groups (Group 1 "NPC", group 2 "Intermediate", group 3 "Neurons", and group 4 "Others" clusters). **(B)** List of top 30 ranked genes in each cluster. **(C)** Separated UMAP and density plot of (i) single, (ii) fused and (iii) connected organoid. **(D)** Relative number of cells in each cluster. **(E)** Pseudo-time differentiation analysis and clustering by the same label of clustering. **(F)** UMAP plot of GRIN2B, ANKRD36, MACF1, and TIA1, which were expressed more in the cells in cluster 8. **(G)** Relative gene expression plot of GRIN2B, ANKRD36, MACF1, and TIA1 in cluster 8 of single, fused, and connected organoids. Expression of the genes in cluster 8 of connected organoids was higher than that of single and fused organoids. P = 0.0001 (single/connected), 0.0027 (fused/connected, GRIN2B); 0.0014 (single/connected), 0.0003 (fused/connected, ANKRD36); 5.2e-5 (single/connected), 0.0008 (fused/connected, MACF1); 0.0084 (single/fused), 0.0002 (single/connected), 8.9e-11 (fused/connected, TIA1). n = 229, 311, 215 cells (GRIN2A); 228, 149, 340 (ANKRD36); 242, 173, 396 (MACF1); 259, 170, 359 (TIA1).**p*<0.05; one-way ANOVA with Tukey's multiple comparison test. Data are presented as mean values ± SD.

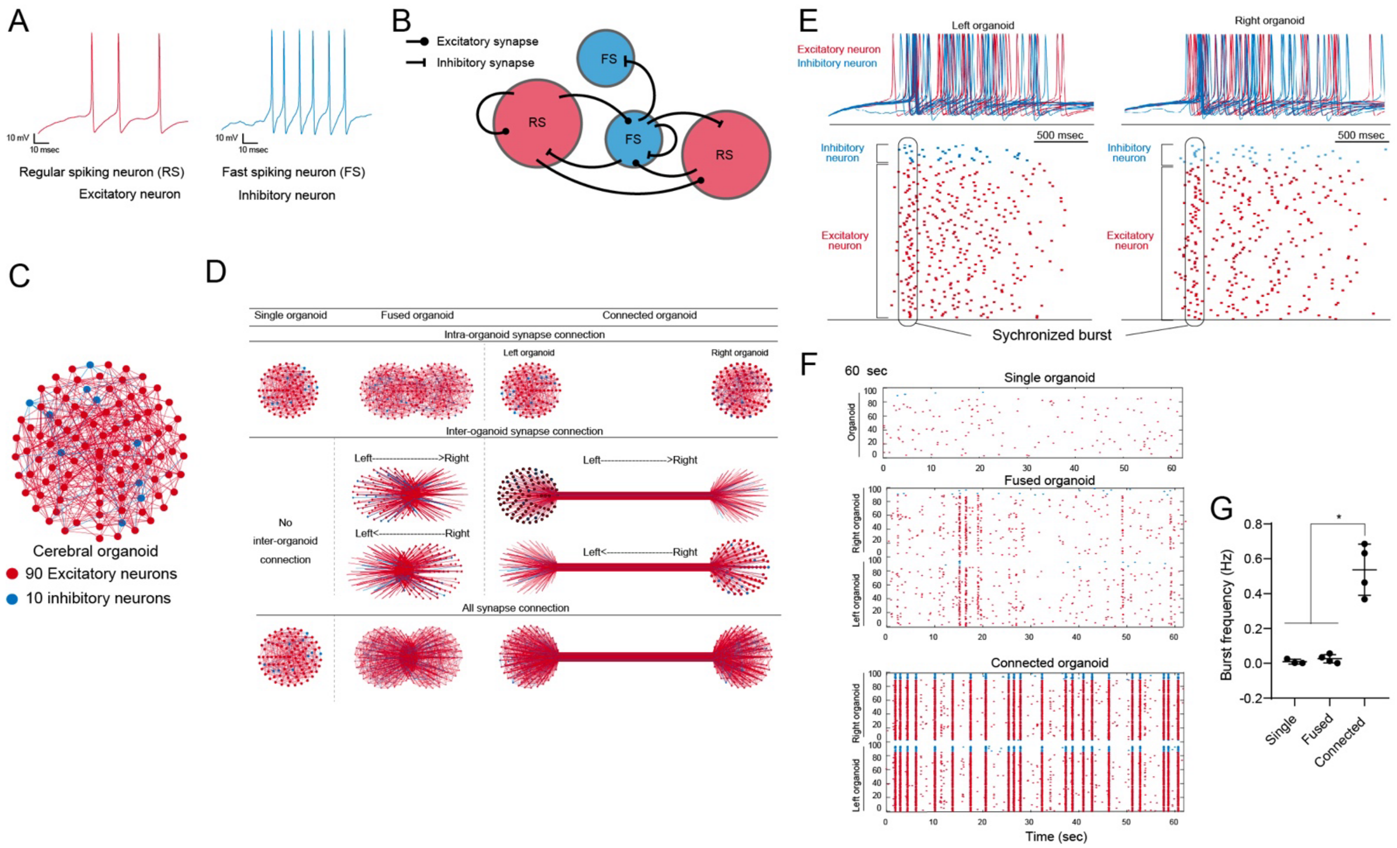

Fig. S7 | In silico connected organoid simulation.

(A) Regular spiking neuron (RS) and fast spiking neuron (FS) by Hodgkin-Huxley model. **(B)** Simplified model of neuronal networks by RS and FS. **(C)** modeled neuronal networks in cerebral organoid. A cerebral organoid model consists of 90 RS neurons and 10 FS neurons. **(D)** Inter- and intra- organoid connection in single, fused, and connected organoids. **(E)** Raw amplitude and spikes by the model. **(F)** Raster plot of single, fused, and connected organoids for 60 sec. **(G)** Quantification of burst frequency. n = 4 simulations. P = 0.0002 (single/connected); 0.0001 (fused/connected). *p<0.05; one-way ANOVA with Tukey's multiple comparison test. Data are presented as mean values ± SD.

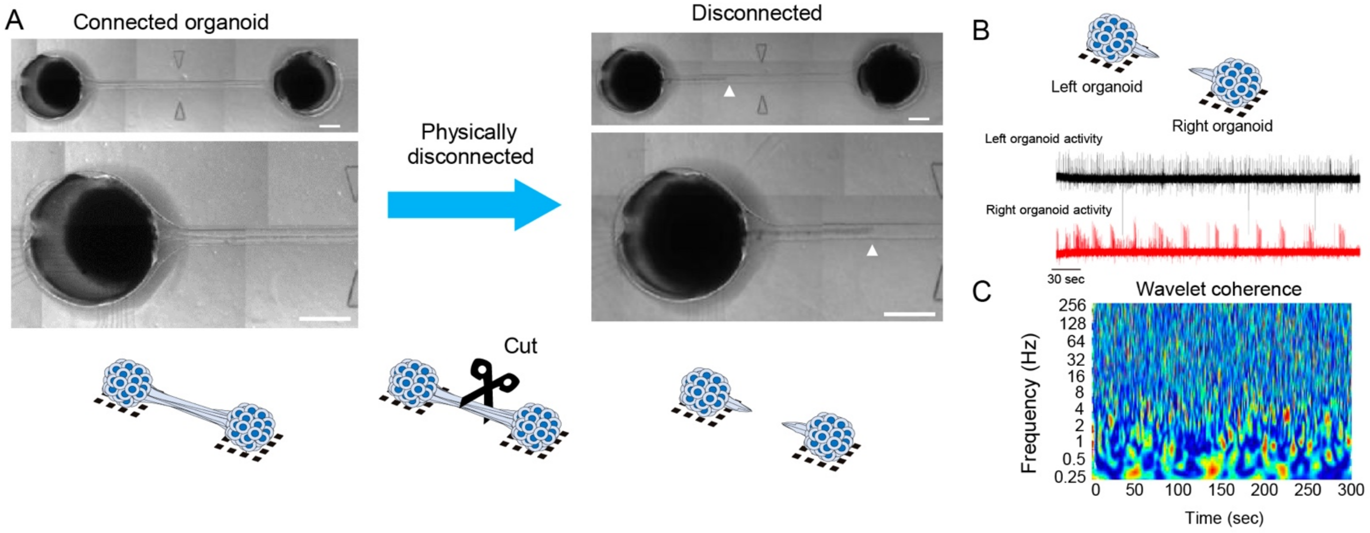

Fig. S8 | Physically disconnected organoids lose synchronized activity.

(A) An axon bundle between connected organoids was physically cut and disconnected (white arrow). Scale bar: 500 μm**. (B)** After cutting the axon bundle, synchronized activity was completely lost. **(C)** Wavelet coherence indicated little or no synchronized activity in the disconnected organoid.

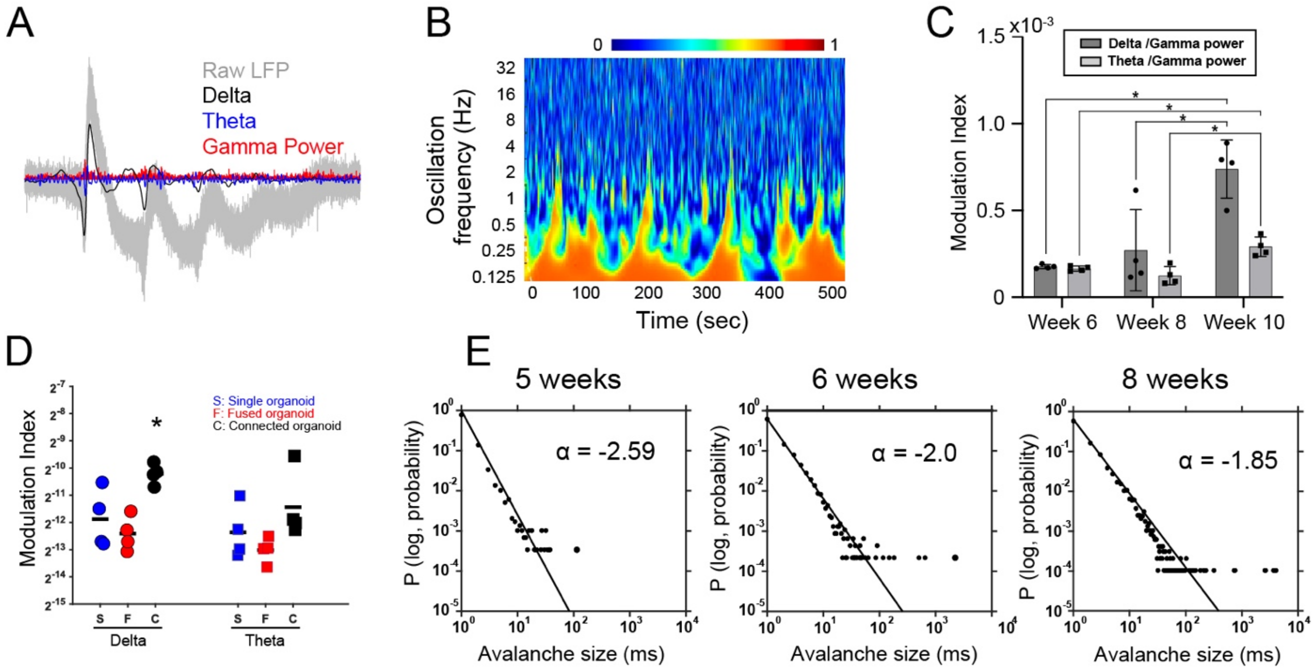

Fig. S9 | Theta-band oscillation, phase-amplitude coupling, and neuronal avalanches in 30HU-002-derived organoids.

(A) Raw LFP plot. **(B)** Wavelet coherence between the two organoids exhibited synchronous activity in the theta band frequency. **(C)** Modulation index of PAC in delta-phase/gamma-power and theta-phase/gamma-power of the connected organoids cultured for 6, 8, and 10 weeks. n = 4 organoids. P = 0.0026; 0.008 (Delta/Gamma power); 0.0086; 0.0015(Theta/Gamma power). **(D)** Delta-phase/gamma-power and theta-phase/gamma-power PAC modulation index of single, fused, and connected organoids. n = 4 organoids. P = 0.0298. **(E)** The log plot of neuronal avalanche size and probability at 5, 6, and 8 weeks of culture. n = 16. *p<0.05; One-way ANOVA with Tukey's multiple comparison test. Data are presented as mean values ± SD.

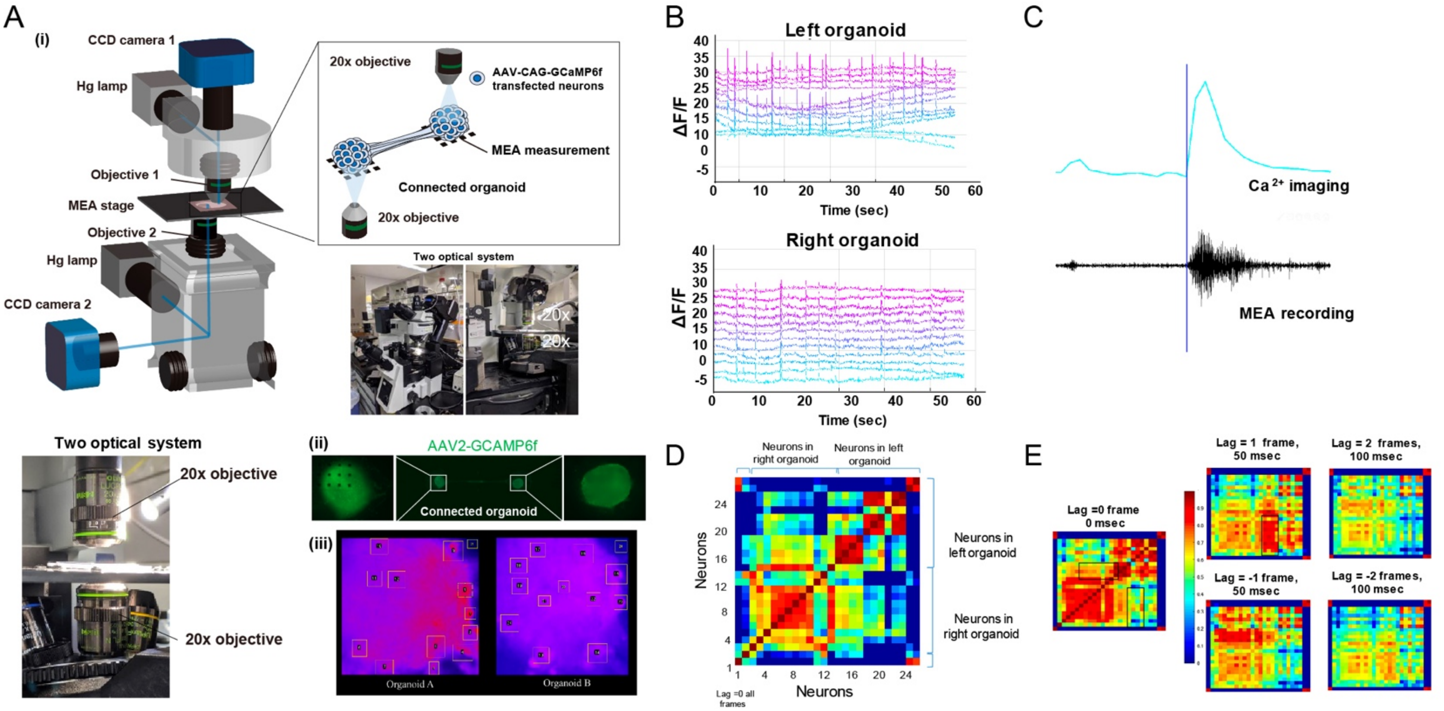

Fig. S10 | Simultaneous measurement of Ca^{2+} transient and electrical activity.
(A) (i) Optical set-up for simultaneous Ca^{2+} imaging and MEA recording of the connected organoids. **(ii, iii)** Ca^{2+}-reporter gene (GCaMP6f) was transiently transfected to the connected organoids 3 days prior to the measurement. Then, time-series images were captured with a custom-made microscope setup while LFP activity from MEA was acquired simultaneously. **(B)** The firing patterns of the cells in the connected organoids are shown by a trace image of the calcium response at 7 weeks of differentiation. **(C)** Plot of both Ca^{2+} activity and MEA signal. Two types of signals corresponded to each other. Consistency of Ca^{2+} imaging and MEA recording during a burst activity. **(D, E)** Correlation matrix from 12 neurons on left organoid and 12 neurons on right organoid is shown for 10 sec measuring time. 2 negative control ROIs serve as the reference. Ca^{2+} activity was strongly correlated within each organoid. Ca^{2+} activity was more strongly correlated between the organoids when the signals were shifted 50 msec.

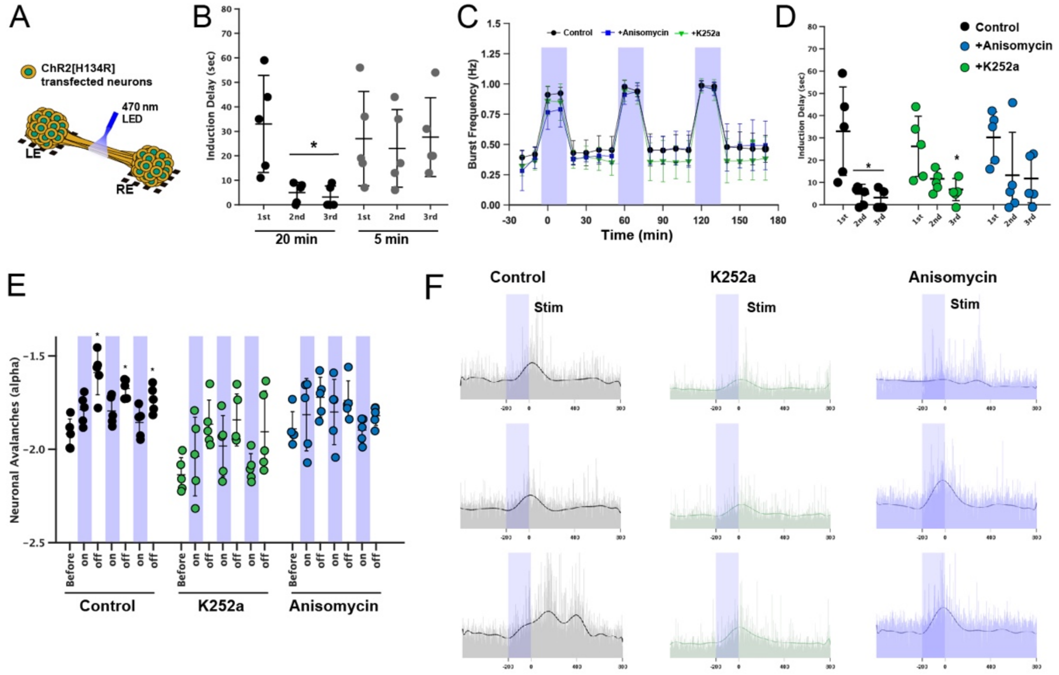

Fig. S11 | Potentiation of the connected organoids generated from 30HU-002 by optogenetic stimulation.

(A) Optogenetic setup for stimulation of the axon bundle with a 470-nm laser. **(B)** The delay from the start of light stimulation to the induction of burst frequency was significantly reduced during the second and third attempts compared to that during the first attempt when the connected organoids were stimulated for 20 min. n = 5 independent experiments from 2 organoids. P = 0.008; 0.0052 (2nd and 3rd relative to 1st, 20 min). **(C)** Time series of burst frequency in the presence of K252a or anisomycin. n = 5 independent experiments from 2 organoids. **(D)** The delay of burst frequency induction of the connected organoids in the presence of K252a or anisomycin. n = 5 independent experiments from 2 organoids. P = 0.008; 0.0052 (control); 0.011 (K252a). **(E)** Probability slope of neuronal avalanches upon K252a or anisomycin treatment. n = 5 independent experiments from 2 organoids. P = 1.3e-5; 0.011; 0.0184. **(F)** Overlayed power histograms of evoked bursts and kernel density estimation (line) in the presence of K252a and anisomycin. *p<0.05; one-way ANOVA with Tukey's multiple comparison test. Data are presented as mean values ± SD.

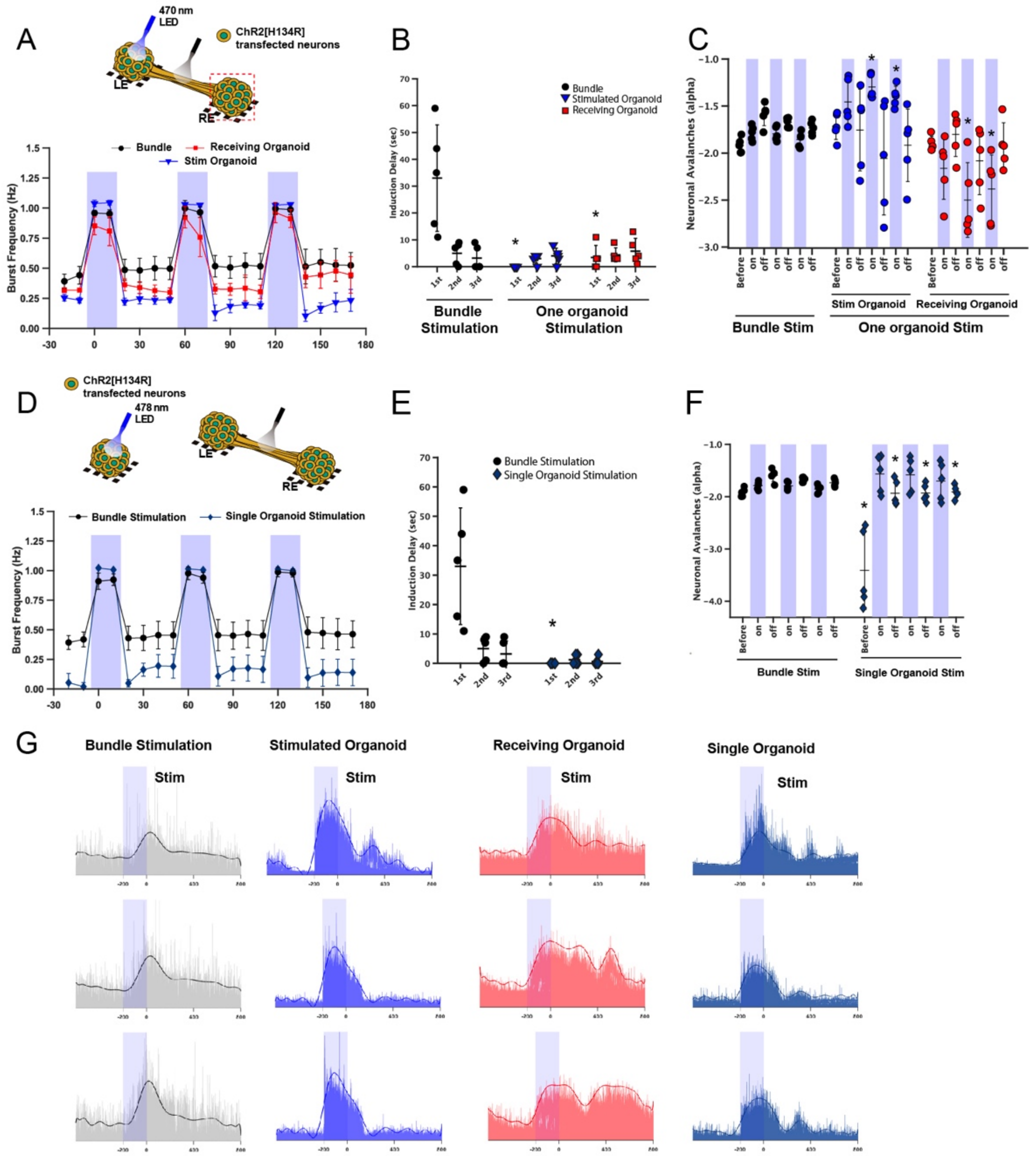

Fig. S12 | Comparing optogenetic stimulation of organoid stimulation with bundle stimulation and single organoids with connected organoids (30HU-002-derived).

(A) Burst frequency of connected organoids upon optogenetic stimulation of either the axon bundle (black) or organoid (blue) and the corresponding receiving organoid following organoid stimulation (red). n = 3. **(B)** Compared to axon bundle stimulation, organoid stimulation of connected organoids resulted in low induction delay that was not significantly changed following the second and third attempt. n = 5 independent experiments from 2 organoids. P = 0.0021 (stimulated organoid); 0.048 (receiving organoid). **(C)** Probability slope of neuronal avalanches following bundle stimulation and organoid stimulation in connected organoids. n = 5 independent experiments from 2 organoids. P = 0.019; 0.0178 (stimulated organoid); 0.0018; 0.0074 (receiving organoid). **(D)** Burst frequency of single organoid and connected organoid (bundle stimulation) following optogenetic stimulation. n = 5 independent experiments from 2 organoids. **(E)** Comparison of induction delay following optogenetic stimulation of either axon bundles in connected organoids or of single organoids. As seen in (B), stimulation of single organoid also resulted in low induction delay that was not significantly changed following the second and third attempt. n = 5 independent experiments from 2 organoids. P = 0.0059. **(F)** Probability slope of neuronal avalanches following stimulation of single organoids or axon bundle of connected organoid. n = 5

independent experiments from 2 organoids. P = 0.0022; 0.0216; 0.0114; 0.0235 (single organoid stimulation). **(G)** Overlayed power histograms of evoked bursts and kernel density estimation (line) upon optogenetic stimulation of single organoids and different regions of connected organoids. *$p<0.05$; one-way ANOVA with Tukey's multiple comparison test. Data are presented as mean values ± SD.

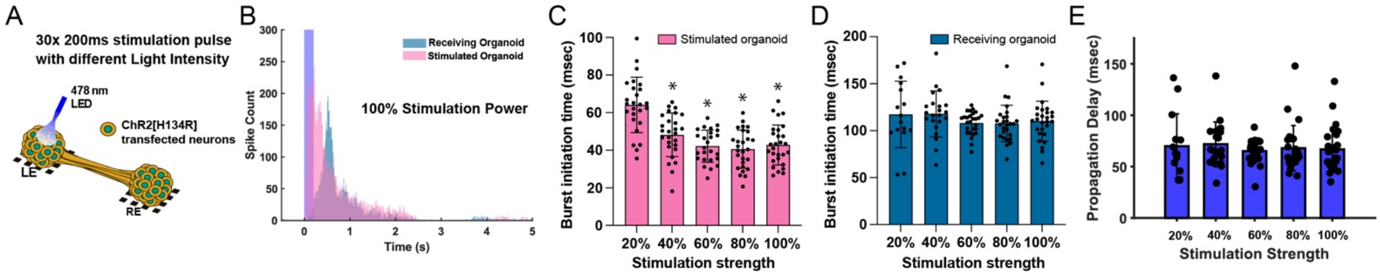

Fig. S13 | Optogenetic stimulation of connected organoids derived from 30HU-002 with different light intensities.

(A) Schematic of the optogenetic stimulation. Light stimulation was applied to one organoid at different light intensities. 100% stimulation strength corresponds to approximately 2.7mW, 80% to 2.25mW, 60% to 1.8mW, 40% to 1.35mW, 20% to 0.9mV. **(B)** Spike count of the stimulated and receiving organoid after receiving 100% power stimulation. Blue shading indicates the stimulation period. **(C)** Burst initiation time of stimulated organoid decreased with increasing stimulation strength. n = 27 stimulations. P = 1.2e-5, 2.0e-9, 7.1e-11, 2.0e-9 (40%-100%, relative to 20%) **(D)** Burst initiation time of receiving organoid did not change following different stimulation strength. n = 27 stimulations. **(E)** Propagation delay between stimulated organoid and receiving organoid upon different stimulation strength. n = 27 stimulations. Not statistically significant; One-way ANOVA with Tukey's multiple comparison test. Data are presented as mean values ± SD.

Table S1. Real-time PCR primers

Gene	Forward primer 5'-3'	Reverse primer 5'-3'
**DCX**	TCCCGGATGAATGGGTTGC	GCGTACACAATCCCCTTGAAGTA
**FOXG1**	*AGGAGGGCGAGAAGAAGAAC*	TCACGAAGCACTTGTTGAGG
**GAD1**	GCGGACCCCAATACCACTAAC	CACAAGGCGACTCTTCTCTTC
**GAD2**	TGGCGTTTCTGCAAGATGTTA	TTGGTCTGCCAATTCCCAATTAT
**GRIA1**	TGATGGAAAATACGGAGCCC	CTTCCCGGACCAAAGTGATAG
**LHX6**	GGGCGCGTCATAAAAAGCAC	TGAACGGGGTGTAGTGGATGT
**MAP2**	CAGGAGACAGAGATGAGAATTCC	CAGGAGTGATGGCAGTAGAC
**NESTIN**	CAACAGCGACGGAGGTCTC	GCCTCTACGCTCTCTTCTTTGA
**NKX2.1**	CGACTCCGTTCTCAGTGTCTGA	CCTCCATGCCCACTTTCTTG
**PAX6**	ACCCATTATCCAGATGTGTTTGCCCGAG	ATGGTGAAGCTGGGCATAGGCGGCAG
**SATB1**	GATCATTTGAACGAGGCAACTCA	TGGACCCTTCGGATCACTCA
**SATB2**	GACAGTGGCCGACATGCTAC	AGGCAAGTCTTCCAACTTTGAA
**SOX2**	GCCGAGTGGAAACTTTTGTCG	GCAGCGTGTACTTATCCTTCTT
**TBR1**	ATGGGCAGATGGTGGTTTTA	GACGGCGATGAACTGAGTCT
**TBR2**	CACCGCCACCAAACTGAGAT	CGAACACATTGTAGTGGGCAG
**TUBB3**	GGCCAAGGGTCACTACACG	GCAGTCGCAGTTTTCACACTC
**VGLUT1**	CAGAGTTTTCGGCTTTGCTATTG	GCGACTCCGTTCTAAGGGTG
**DLX5**	TTCCAAGCTCCGTTCCAGAC	GAATCGGTAGCTGAAGACTCG
**BCL11A**	CGCCAGAGGATGACGATTGTT	CCAGGCGTGGGGATTAGAG
**BCL11B**	GGTGCCTGCTATGACAAGG	GGCTCGGACACTTTCCTGAG
**GAPDH**	TGTGGGCATCAATGGATTTGG	ACACCATGTATTCCGGGTCAAT

Table S2. Key resource

Reagent and resource	Source	Identifier
**Cell line**		
Human iPSC 409B2	Riken	HPS0076
Human iPSC 30HU-002	iXCell	N.A.
AAVpro 293T	Takara	N.A.
**Plasmid**		
AAV-CAG-hChR2[H134R]-tdTomato	Addgene	#28017
pAAV-CAG-ArchT-GFP	Addgene	#29777
AAV-CAG-EGFP	Addgene	#28014
AAV-CAG-Kaede-WPRE	This paper	N.A.
pAAV.CAG.GCaMP6f.WPRE.SV40	Addgene	#100836
PX458-AAVS1	Addgene	#113194
AAVS1-Pur-CAG-EGFP	Addgene	#80945
AAVS1-Pur-CAG-mCherry	Addgene	#80946
pME-BrainbowTEC	Addgene	#82405
AAVS1-Pur-CAG-BrainbowTEC	This paper	N.A.
**AAV**		
[AAV-retro] AAV-CAG-hChR2[H134R]-tdTomato	Addgene	#28017-AAVrg
[AAV1] pAAV-ArchT-GFP	Addgene	#29777-AAV1
[AAV1] pAAV.CAG.GCaMP6f.WPRE.SV40	Addgene	#28014-AAV1
[AAV1] AAV-CMV-Cre	This paper	N.A.
[AAV2] AAV-CAG-Kaede-WPRE	This paper	N.A.

Supplementary Reference

1 Pospischil, M. *et al.* Minimal Hodgkin-Huxley type models for different classes of cortical and thalamic neurons. *Biol Cybern* **99**, 427-441 (2008). https://doi.org/10.1007/s00422-008-0263-8

2 Khoyratee, F., Grassia, F., Saïghi, S. & Levi, T. Optimized Real-Time Biomimetic Neural Network on FPGA for Bio-hybridization. *Frontiers in Neuroscience* **13** (2019). https://doi.org/10.3389/fnins.2019.00377

3 Destexhe, A., Mainen, Z. F. & Sejnowski, T. J. Synthesis of models for excitable membranes, synaptic transmission and neuromodulation using a common kinetic formalism. *J Comput Neurosci* **1**, 195-230 (1994). https://doi.org/10.1007/BF00961734

4 Destexhe, A., Rudolph, M., Fellous, J. M. & Sejnowski, T. J. Fluctuating synaptic conductances recreate in vivo-like activity in neocortical neurons. *Neuroscience* **107**, 13-24 (2001). https://doi.org/10.1016/s0306-4522(01)00344-x

5 Wolf, F. A., Angerer, P. & Theis, F. J. SCANPY: large-scale single-cell gene expression data analysis. *Genome Biology* **19**, 15 (2018). https://doi.org/10.1186/s13059-017-1382-0

6 Chen, Y. *et al.* Chemical Control of Grafted Human PSC-Derived Neurons in a Mouse Model of Parkinson's Disease. *Cell Stem Cell* **18**, 817-826 (2016). https://doi.org/https://doi.org/10.1016/j.stem.2016.03.014
