# Supplementary material for: Complex activity and short-term plasticity of human cerebral organoids reciprocally connected with axons
Source: Nat Commun. 2024 Apr 10;15:2945. doi: 10.1038/s41467-024-46787-7 (PMC11006899; doi:10.1038/s41467-024-46787-7)
Supplement: Supplementary file 2 — Reporting Summary [file 41467_2024_46787_MOESM2_ESM.pdf]

nature portfolio

Corresponding author(s): Yoshiho Ikeuchi

Last updated by author(s): 2024/02/23

Reporting Summary

Nature Portfolio wishes to improve the reproducibility of the work that we publish. This form provides structure for consistency and transparency in reporting. For further information on Nature Portfolio policies, see our Editorial Policies and the Editorial Policy Checklist.

Statistics

For all statistical analyses, confirm that the following items are present in the figure legend, table legend, main text, or Methods section.

n/a	Confirmed	
☐	☒	The exact sample size (*n*) for each experimental group/condition, given as a discrete number and unit of measurement
☐	☒	A statement on whether measurements were taken from distinct samples or whether the same sample was measured repeatedly
☐	☒	The statistical test(s) used AND whether they are one- or two-sided<br>*Only common tests should be described solely by name; describe more complex techniques in the Methods section.*
☒	☐	A description of all covariates tested
☐	☒	A description of any assumptions or corrections, such as tests of normality and adjustment for multiple comparisons
☐	☒	A full description of the statistical parameters including central tendency (e.g. means) or other basic estimates (e.g. regression coefficient) AND variation (e.g. standard deviation) or associated estimates of uncertainty (e.g. confidence intervals)
☐	☒	For null hypothesis testing, the test statistic (e.g. *F*, *t*, *r*) with confidence intervals, effect sizes, degrees of freedom and *P* value noted<br>*Give P values as exact values whenever suitable.*
☒	☐	For Bayesian analysis, information on the choice of priors and Markov chain Monte Carlo settings
☒	☐	For hierarchical and complex designs, identification of the appropriate level for tests and full reporting of outcomes
☒	☐	Estimates of effect sizes (e.g. Cohen's *d*, Pearson's *r*), indicating how they were calculated

Our web collection on statistics for biologists contains articles on many of the points above.

Software and code

Policy information about availability of computer code

Data collection	MEA data were aquired by MED64 system (Alpha MED Scientific) and electrical signals from all 64 electrodes were recorded for 5-30 min at 37°C at 20,000 Hz sampling rate. The recording noise was eliminated by band-pass filer between 0.1-10000 Hz during the measurement. scRNAseq were perforemd with 10x Genomics Chromium single-cell RNA-seq library preparation, according to the manufacturer's protocol. the library was sequencing with 150-bp paired-end reads on DNBSEQ. Patch-clamp recordings were performed using MultiClamp700B amplifiers (Molecular Devices), filtered at 3 kHz using a Bessel filter and digitized at 20 kHz with Digidata 1550B digitizer (Molecular Devices) with pClamp software. Time-lapse images for Ca imaging were captured at least at a frame rate of 20 fps for 10 min.<br>All images acquired with an Axio Observer inverted microscope with Zeiss ZEN blue and a Nikon confocal microscope (A1R) with NIS elements.
Data analysis	The MEA recording data was analyzed using MATLAB software (2019a).<br>Source codes for MEA analysis and used for in silico organoid model are available at Mendeley Data (https://dx.doi.org/10.17632/vcf5f2k5yb.1), and github (https://github.com/Ceramic-Blue-Tim/in-silico-connected-organoid-model; https://github.com/utokyoIkeuchilab/complex_activity_data_analysis).<br>scRNA-seq data were processed using Cell Ranger analysis pipeline v3 and Scampy.<br>All patch clamping recording data were implemented using the Matlab (2019a) and Origin Pro 2017.<br>Ca imaging data analysis was carried out using MATLAB (2019a).<br>Flowcytometry data were analyzed by Flowjo.<br>Image analysis were performed with Fiji and NIS elements.

For manuscripts utilizing custom algorithms or software that are central to the research but not yet described in published literature, software must be made available to editors and reviewers. We strongly encourage code deposition in a community repository (e.g. GitHub). See the Nature Portfolio guidelines for submitting code & software for further information.

Data

Policy information about availability of data

All manuscripts must include a data availability statement. This statement should provide the following information, where applicable:

- Accession codes, unique identifiers, or web links for publicly available datasets
- A description of any restrictions on data availability
- For clinical datasets or third party data, please ensure that the statement adheres to our policy

All data in this paper is available within the article, supplementary file or source data. scRNA-Seq data (GEO: GSE190729) used in this study have been deposited to NCBI. Other data used in this study are available from corresponding author upon request.

Research involving human participants, their data, or biological material

Policy information about studies with human participants or human data. See also policy information about sex, gender (identity/presentation), and sexual orientation and race, ethnicity and racism.

Reporting on sex and gender	Not applicable
Reporting on race, ethnicity, or other socially relevant groupings	Not applicable
Population characteristics	Not applicable
Recruitment	Not applicable
Ethics oversight	Not applicable

Note that full information on the approval of the study protocol must also be provided in the manuscript.

Field-specific reporting

Please select the one below that is the best fit for your research. If you are not sure, read the appropriate sections before making your selection.

[x] Life sciences [ ] Behavioural & social sciences [ ] Ecological, evolutionary & environmental sciences

For a reference copy of the document with all sections, see nature.com/documents/nr-reporting-summary-flat.pdf

Life sciences study design

All studies must disclose on these points even when the disclosure is negative.

Sample size	No statistical methods were used to predetermine sample size. All sample size was determined by the number of organoid samples available.
Data exclusions	We excluded organoids that had no electrical activity recorded.
Replication	Reproducibility of experiments were confirmed; Key findings were replicated across independent experiments.
Randomization	Randomization is not applicable in this study. All data were acquired by machines and analyzed by custom scripts. All organoids used in the study were treated equally and generated from the same protocol.
Blinding	Blinding is not applicable. All data sets were acquired by machines and analyzed by custom scripts without assumption of a trend. All organoids used in the study were treated equally and generated from the same protocol.

Reporting for specific materials, systems and methods

We require information from authors about some types of materials, experimental systems and methods used in many studies. Here, indicate whether each material, system or method listed is relevant to your study. If you are not sure if a list item applies to your research, read the appropriate section before selecting a response.

Materials & experimental systems

n/a	Involved in the study
[ ]	[x] Antibodies
[ ]	[x] Eukaryotic cell lines
[x]	[ ] Palaeontology and archaeology
[x]	[ ] Animals and other organisms
[x]	[ ] Clinical data
[x]	[ ] Dual use research of concern
[x]	[ ] Plants

Methods

n/a	Involved in the study
[x]	[ ] ChIP-seq
[ ]	[x] Flow cytometry
[x]	[ ] MRI-based neuroimaging

Antibodies

Antibodies used	The primary antibodies were mouse anti-neuron-specific βIII tubulin (Biolegend, 801202, 1:1200), rabbit anti-neuron-specific βIII tubulin (Sigma ZooMAb, ZRB1140, 1:200), rabbit anti-PAX6 (Wako, 015-27293, 1:500), mouse anti-human GAD67 (Santa Cruz, sc-28376, 1:100), rabbit anti-human VGLUT1 (Sigma ZooMAb, ZRB2374, 1:200), rat anti-human CTIP2 (Abcam, ab18465 1:200), mouse anti-Tau1 (Merck, MAB3420, 1:1000), and rabbit anti-synapsin (Merck, AB1543, 1:500). The secondary antibodies were Alexa Fluor 568 goat anti-rabbit IgG (H+L) (Thermo Fisher Scientific, A11036), Alexa Fluor 488 goat anti-mouse IgG (H+L) (Thermo Fisher Scientific, A11029), and Alexa Fluor 488 goat anti-rat IgG H&L (Abcam, ab150165).
Validation	All antibodies used were commercially purchased and were validated for their respective application by their manufacturer.

Eukaryotic cell lines

Policy information about cell lines and Sex and Gender in Research

Cell line source(s)	Human iPSC 409B2 (Riken HPS0076), Human iPSC 30HU-002 (iXCell), AAVpro 293T (Takara)
Authentication	Human iPSCs were characterized by pluropotency markers and karyotyped by a qPCR kit (hPSC Genetic Analysis Kit, STEMCELL Technologies)
Mycoplasma contamination	All cell lines tested negative for mycoplasma.
Commonly misidentified lines (See ICLAC register)	No misidentified cell line was used.

Plants

Seed stocks	Not applicable
Novel plant genotypes	Not applicable
Authentication	Not applicable

Flow Cytometry

Plots

Confirm that:

- [x] The axis labels state the marker and fluorochrome used (e.g. CD4-FITC).
- [x] The axis scales are clearly visible. Include numbers along axes only for bottom left plot of group (a 'group' is an analysis of identical markers).
- [x] All plots are contour plots with outliers or pseudocolor plots.
- [x] A numerical value for number of cells or percentage (with statistics) is provided.

Methodology

Sample preparation	Cells were suspended with PBS + 3% BSA. Cell suspension were filtered through by 70 um filter.

Instrument	BD FACSMelody
Software	Data were acquired by BD FACS Chorus and data were analyzed by Flowjo.
Cell population abundance	10000 neurons were analyzed and sorted per sample.
Gating strategy	The cells were then separated by gating the main populations using SSC/FSC.

☐ Tick this box to confirm that a figure exemplifying the gating strategy is provided in the Supplementary Information.
